# Watershed-Induced Limnological and Microbial Status in Two Oligotrophic Andean Lakes Exposed to the Same Climatic Scenario

**DOI:** 10.3389/fmicb.2018.00357

**Published:** 2018-03-05

**Authors:** Alex Echeverría-Vega, Guillermo Chong, Antonio E. Serrano, Mariela Guajardo, Olga Encalada, Victor Parro, Yolanda Blanco, Luis Rivas, Kevin C. Rose, Mercedes Moreno-Paz, José A. Luque, Nathalie A. Cabrol, Cecilia S. Demergasso

**Affiliations:** ^1^Centro de Biotecnología, Universidad Católica del Norte, Antofagasta, Chile; ^2^Departamento de Ciencias Geológicas, Universidad Católica de Norte, Antofagasta, Chile; ^3^Centro de Investigación Científica y Tecnológica para la Minería, Antofagasta, Chile; ^4^Department of Molecular Evolution, Centro de Astrobiología (CSIC-INTA), Madrid, Spain; ^5^Department of Biological Sciences, Rensselaer Polytechnic Institute, Troy, NY, United States; ^6^Centro de Investigación Tecnológica del Agua en el Desierto (CEITSAZA), Universidad Católica del Norte, Antofagasta, Chile; ^7^Carl Sagan Center, SETI Institute, Mountain View, CA, United States; ^8^Space Science Division, NASA Ames Research Center, Moffett Field, CA, United States

**Keywords:** oligotrophic lakes, microbial communities, glacial melting, 16srRNA gene sequencing, watershed influence

## Abstract

Laguna Negra and Lo Encañado are two oligotrophic Andean lakes forming part of the system fed by meltwater from distinct glacial tongues of the Echaurren glacier in central Chile, which is in a recession period. The recent increase in temperature and decline in precipitation have led to an increase of glacial meltwater and sediments entering these lakes. Although the lacustrine systems are also hydrogeologically connected, the limnology of the lakes is strongly controlled by the surface processes related to the respective sub-watersheds and hydrology. Watershed characteristics (area and length, slope, lithology, resistance to erosion, among others) affect the chemical and physical characteristics of both lakes (e.g., nutrient concentration and turbidity). We studied physical and chemical variables and performed 16S rRNA amplicon sequencing to determine the specific microbial signature of the lakes. The transparency, temperature, turbidity and concentrations of chlorophyll-a, dissolved organic matter, nutrients and the total number of cells, revealed the different status of both lakes at the time of sampling. The predominant bacterial groups in both lakes were Proteobacteria, Verrucomicrobia, and Bacteroidetes. Interestingly, the contribution of phototrophs was significantly higher in LN compared to LE (13 and 4% respectively) and the major fraction corresponded to Anoxygenic Phototrophs (AP) represented by Chloroflexi, Alpha, and Betaproteobacteria. Multivariate analyses showed that the nutrient levels and the light availability of both lakes, which finally depend on the hydrological characteristics of the respective watersheds, explain the differential community composition/function. The abundance of a diverse photoheterotrophic bacterioplankton community suggests that the ability to utilize solar energy along with organic and inorganic substrates is a key function in these oligotrophic mountain lakes.

## Introduction

Glaciers are one of the reservoirs of freshwater on the planet and are sensitive to small changes in global temperature. Glacial evolution depends on the balance between ice accumulation and ablation rate (Bennett and Glasser, 2009). The increased melting of High Andean glaciers due to rising temperatures has resulted in a sustained decrease in their volume over the last 50 years (Rivera et al., 2002; Carrasco et al., 2005; Bown and Rivera, 2007; Le Quesne et al., 2009; Fernández and Mark, 2016) and in important hydrological changes downstream (Vicuña et al., 2010; Pizarro et al., 2013, 2016), which has greatly impacted the physical and chemical characteristics of glacially-fed lakes (Gunn et al., 2001; Bown and Rivera, 2007; Rose et al., 2014; Pizarro et al., 2016). The trophic status of these lakes may be related to higher nutrient influx (eutrophication) or to climate change (e.g., higher temperatures, light, stratification, among other factors) (Hakanson and Jansson, 2002; Blass et al., 2007; McKay et al., 2008), depending on the limiting factor in the aquatic ecosystems. Variations in glacial outflow water temperature, volume, timing, nutrient concentrations, and optically active substances (Gunn et al., 2001; Saros et al., 2010; Pizarro et al., 2016) can also lead to significant changes in the lake ecosystems like changes in their trophic status, stratification, the community composition and the increasing in phytoplankton mortality (van Duin et al., 1992; Sommaruga, 2015). In addition, paleolimnological research has demonstrated that oligotrophic lakes constitute highly sensitive systems that record past environmental changes, such as natural climate oscillations, anthropogenic contamination and catastrophic events triggered by earthquakes (Luque and Julià, 2002; Julià and Luque, 2006; Feeley et al., 2012; Xu, 2017).

Variations in glacially-fed lakes may alter microbial communities in these ecosystems in important ways. Furthermore, because microorganisms play essential roles in globally important biogeochemical cycles and processes, it is critical to understand environmental factors that contribute to patterns in microbial communities. In addition, microbial cells represent the major fraction of the particulate organic carbon in oligotrophic lakes. Microorganisms respond rapidly to variations in nutrient concentration and availability through changes in the structure and diversity of microbial communities (Yannarell and Triplett, 2005; Newton et al., 2011; Logue et al., 2012; Slemmons et al., 2013; Niño-García et al., 2016). The presence or absence and abundance of microbial taxa depend on both resource availability and mortality factors, which lead to distribution patterns at both micro and macro levels (Green and Bohannan, 2006). Their occurrence and organization are closely related to the physicochemical characteristics of the water (Marion et al., 2003; Møller et al., 2013), geographical factors (Catalan et al., 2009; Sommaruga and Casamayor, 2009), and the influence of the local environment (Sommaruga and Casamayor, 2009; Sarmento et al., 2015).

We used 16S rRNA amplicon sequencing to determine the composition and to predict metabolic traits of the bacterial communities present in two recently separated Andean lakes, Laguna Negra and Lo Encañado. Both lakes are fed by Echaurren glacier, which has shown a sustained negative mass-balance between 1993 and 2012 (Mernild et al., 2015). The negative mass-balance of glaciers determines changes in the hydrological characteristics associated with the snow melting processes (Center for Climate and Resilience Research (CR)2, 2015), and strongly controls the streamflow recorded in the drainage system which is feeding the lacustrine systems. Therefore, fluctuations of streamflow could trigger changes in the microbial and limnological characteristics since the water input finally controls the trophic status of the lakes and their stratification (Cumming, 2003; Soranno et al., 2010). Once the comparison highlighted the differences between the community structures of both lakes fed by the Echaurren glacier, we identified factors involved in shaping the observed differences. The comparison among closely-related oligotrophic lakes under the same environmental condition allows us to enhance understanding of the limnological response to climate changes.

## Materials and methods

### Geological setting of studied site and sampling

**Figure 1:**
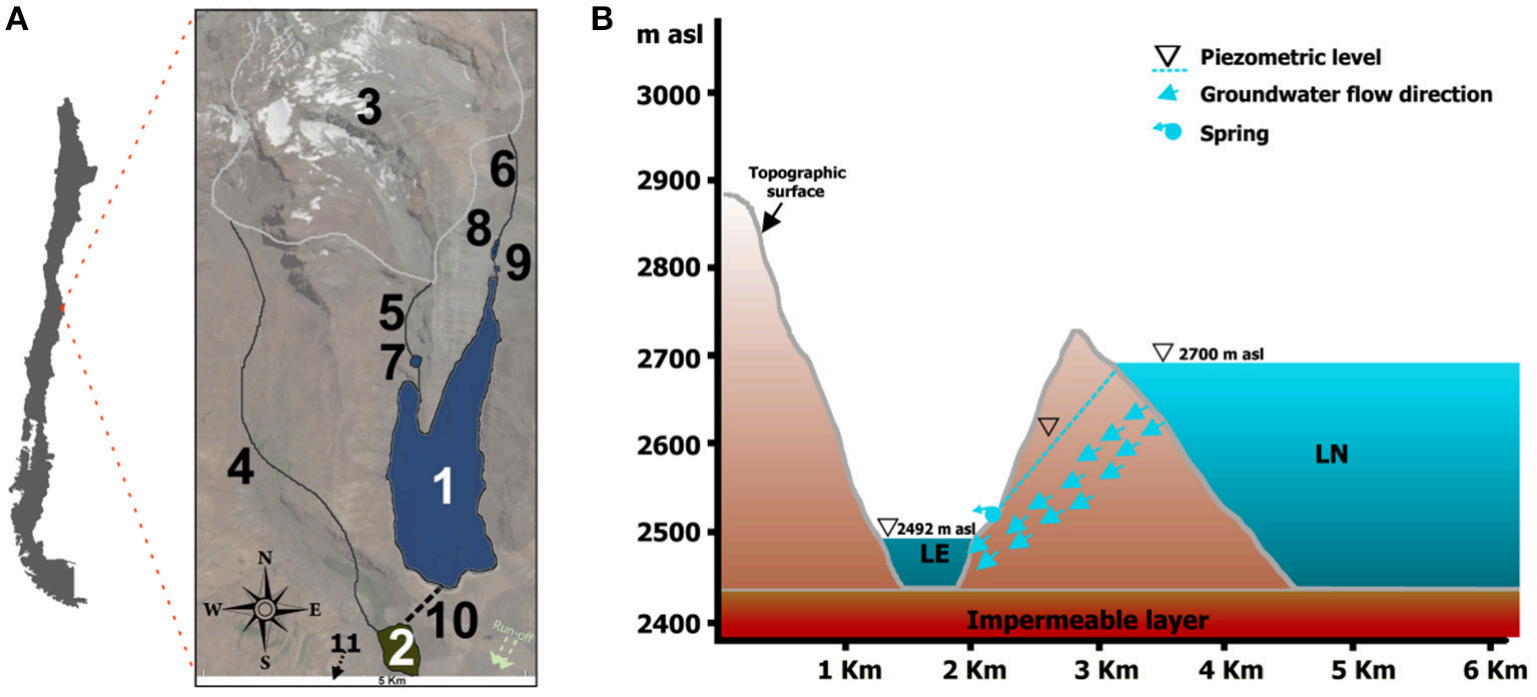
**(A)** Map location of the lake system with geographical characteristics of the area. Significant details shown: (1) Laguna Negra; (2) Lo Encañado; (3) Echaurren glacier; (4) Meltwater flow feeding Lo Encañado; (5) and (6) Meltwater flows feeding Laguna Negra; (7), (8), and (9) Sediment retaining ponds; (10) Former land connection between both lakes (11) Direction of the water flux and location of the Manzanito and Maipo rivers. **(B)** Cross section showing topography and piezometric levels of both lakes.

The climate in central Chile is Mediterranean with cold-humid winters and hot-dry summers with very high solar radiation. Mean annual precipitation is on the order of 550 mm, which is mainly driven by the southern mid-latitude Westwind Drift and the southeast Pacific anticyclone (Aceituno, 1988). The studied lake system is located in the San José de Maipo area, 50 km East of Santiago, in the Central Andes of Chile (Figure 1). It includes lakes Laguna Negra (LN) (33° 39′ S and 70° 7′ W) and Lo Encañado (LE) (33° 40′ S and 70° 8′ W). LN has a surface area of 5.4 km^2^, a maximum depth of 276 m, and is located at 2,700 masl; LE has a surface area of 0.46 km^2^ at 2,492 masl, with a maximum depth of 35 m (Pille, 2013) (Supplementary Presentation 1). Limnological studies carried out by the national water institution of Chile (Dirección General de Aguas, 2014) showed that LN has a stratified water column: the shallow epilimnion (relatively warm waters), the intermediate metalimnion (with gradual decreasing in temperature), and the deep hypolimnion (cold waters). In addition, oxygen concentrations increased toward the bottom of the lacustrine system and were associated with the hypolimnion. Regarding electrical conductivity, salinity, Redox potential, turbidity and response to fluorescent, all parameters were constant in depth. LN and LE were formerly connected by a human-made overflow tunnel, which in the past generated an intermittent water influx from LN to LE. The lake level of LN has substantially decreased and the lakes have been almost completely isolated from each other since at least 2007 (Parro et al., 2016). Moreover, there is a sporadic hydrogeological connection between both lakes as suggested by the evidence of a former small spring along the topographical slope, which separates the lacustrine systems. Groundwater flows from LN (with a piezometric level at 2,700 masl) to LE (with a piezometric level at 2,492 masl) and it is controlled by the potential presence of an impermeable basal level. The conceptual model is shown in Figure 1. LE constitutes the natural discharge of groundwater coming from LN. The catchment area of the lakes is a glacial, mountainous region, ranges up to 4,600 masl and is dominated by Glacial Echaurren. The glacier shows strong interannual mass balance variations due to its sensitivity toward ENSO events, showing a negative mass balance since 1988 (Casassa et al., 2007). Therefore, LN and LE belong to the same hydrogeological scenario, but the catchment areas of the lakes provide different hydrological inputs to the lacustrine systems. LN lies about 200 vertical meters above the eastern flank of the LE (Figure 1), it has a catchment area 4 times larger than the lake (~ 22 km^2^). The catchment is very steep, with high amount of eroded and detrital sediments and very scarce vegetation coverage (small shrubs and grasses). According to previous reports, LN is one of the least productive lakes in Chile, with an average productivity of 0.3 mg C m^−3^ h^−1^ (Cabrera and Montecino, 1987). LE has a very large catchment area compared to the size of the lake (approximately 30 km^2^), which is 60 times larger than the lake. Upstream of the catchment area, the Lo Encañado River flows through the glacial valley into the LE forming a delta (Pille, 2013) which contains a large amount of terrestrial vegetation and algae. The western and eastern flanks are both very steep, starting directly from the lake shore.

We collected historical data available on precipitation and annual mean temperature from meteorological station at San José de Maipo, located 20 km east of LN (33° 36′ S−70° 21′ W and 928 masl; Dirección Meteorológica de Chile, 1991–2012).

In addition, we conducted sampling campaigns of both lakes between December 5th and December 8th, 2012, toward the end of the Southern Hemisphere spring season. We collected water samples and performed measurements in LN on the 5–6th and in LE on the 7–8th of December. To analyze microbial diversity, we collected 100 L of water, approximately 100 m away from the south shore of each lake using an inflatable boat with an electric engine. Samples were taken from the surface and from the water column at depths of 5, 10, and 20 m. We also collected 1 L samples at each sampling station to carry out laboratory-based physicochemical analysis (specified below). Smaller 15 mL samples were preserved in 4% w v^−1^ formaldehyde to determine the total cell number using epifluorescence microscopy.

### Physicochemical analysis

The water samples we took for physicochemical analysis were kept at 4°C while transported to the laboratory for processing. We determined the concentration of iron, nickel, cadmium, aluminum, silicon, zinc, sodium, magnesium, manganese and arsenic by atomic absorption spectroscopy (Rice et al., 2012); chloride, by the Volhard method (Skoog et al., 2014); sulfate by gravimetric analysis (Skoog et al., 2014); and fluoride, acetate, nitrate and tartrate by ionic chromatography (Parro et al., 2011). We used an Orion multi-tester to determine pH, Eh, turbidity, temperature, and conductivity. We measured fluorescent dissolved organic matter (fDOM), turbidity and chlorophyll-a fluorescence (Chl*a*), using a Turner Designs C6 sensor and, ultraviolet radiation attenuation to 305 nm, 320 nm, 380 nm and photosynthetically active radiation (PAR, 400–700 nm) attenuation using a Cosine submersible radiometer (Biospherical Instruments, USA), as previously described by Rose et al. (2014). Water clarity was also measured using a Secchi disc (Lamotte, USA).

### Microbial counts

Bacterial cells were stained with 4′,6-diamidine-2-phenylindole (DAPI) and enumerated using an Olympus IX-81 epifluorescence microscope (Olympus, Japan; Härd et al., 1990). A minimum of 400 cells were counted in 20 fields.

### DNA extraction

We concentrated the 100 L volume samples down to a volume of 250 mL by means of a tangential flow concentrator with a 0.22 μm nitrocellulose Millipore filter (Merck-Millipore, Germany). Microorganisms were collected by filtration of the pre-concentrated water on a nitrocellulose membrane with a 0.22 μm pore size (Merck-Millipore, Germany). The filter was then folded and placed in a tube with a hypertonic lysis buffer solution (25.7% sucrose, 50 mM TRIS-HCl, 40 mM EDTA) and was kept at −20°C for later processing. DNA was obtained using the AllPrep Qiagen RNA/DNA Isolation Kit (Qiagen, USA). The extraction yield and DNA purity were determined by means of gel electrophoresis and UV-Visible spectrophotometry (Nanodrop, Thermo, Germany).

### Sequencing

We amplified the V1–V3 variable region of the 16S rRNA gene by PCR using primers 27f (5′-AGAGTTTGATCCTGGCTCAG-3′; Lane, 1991) and 534r (5′-ATTACCGCGGCTGCTGG-3′; Muyzer et al., 1993) for bacteria and V3-V4 for Archaea using primers 349f (5′-GYGCASCAGKCGMGAAW-3′) and 806r (5′-GGACTACVSGGGTATCTAAT-3′) (Lenchi et al., 2013) with a barcode on the forward primer. PCR was performed using the HotStarTaq Plus Master Mix Kit (Qiagen, USA) under the following conditions: 94°C for 3 min, followed by 28 cycles of 94°C for 30 s, 53°C for 40 s and 72° C for 1 min, after which we performed a final elongation step at 72°C for 5 min. After amplification, we checked PCR products in 2% agarose gel to determine the success of amplification and the relative intensity of the bands. We pooled multiple samples together in equal proportions based on their molecular weight and DNA concentrations. Pooled samples were purified using calibrated Ampure XP beads. Then, we used the pooled and purified PCR product to prepare a DNA library by following the Illumina TruSeq DNA library preparation protocol. Sequencing was performed at MR DNA (www.mrdnalab.com, Shallowater, TX, USA) on a MiSeq, following the manufacturer's guidelines. We then processed the sequence data using a proprietary analysis pipeline (MR DNA) with the following steps: the sequences were depleted of barcodes, then sequences <150 bp and those with ambiguous base calls were removed. Then, sequences were denoised, OTUs generated and chimeras removed. The reads were added to the Sequence Read Archive (SRA) National Center for Biotechnology Information (NCBI), (http://trace.ncbi.nlm.nih.gov/Traces/sra/) Project number PRJNA289691.

### Functional, phylogenetic and taxonomic analysis

We processed the sequences by means of the QIIME (Quantitative Insights Into Microbial Ecology) pipeline (Caporaso et al., 2010b):

We filtered raw sequences based on base quality score, average base content per read and GC distribution in the reads. Reads that did not cluster with other sequences, i.e., singletons (abundance <2) were removed. Chimeras were also removed using the UCHIME program (Edgar et al., 2011). The pre-processed sequences were finally grouped into operational taxonomic units (OTUs) using the clustering program UCLUST at a similarity threshold of 0.97 (Edgar, 2010). We used all of the pre-processed reads to identify the OTUs using QIIME and aligned the representative sequences against the Greengenes core set reference database using PyNAST (Caporaso et al., 2010a). We classified a representative sequence for each OTU using RDP classifier and the Greengenes OTU database. Then, we calculated the alpha-rarefaction by means of the “core_diversity_analysis” command and standardized the number of sequences to the smaller sample size by means of Chao 1 (73,402 sequences). We processed the rarefacted data using the Primer-6 (Primer-E) software (Clarke, 1993) to determine the beta diversity and plot the main coordinates graphs as further detailed. We used Krone interactive graphs (Ondov et al., 2011) for visualization.

### Data processing

We integrated and analyzed the abundance of microorganisms (OTUs), abundance of genes, and the physical and chemical parameters data sets using the Primer-6 (Primer-E) software (Clarke et al., 2014). We analyzed alpha and beta diversity as well as distance and similarity in environmental, biological and functional data. We used the fourth root of the data obtained for genes and OTUs to homogenize the quantities and reduce the dominance effect. We then constructed Bray–Curtis similarity matrices of transformed abundance data. Environmental analyses (physical and chemical) data were normalized, and their Euclidean distance matrices were constructed. We conducted significance tests using the permutational multivariate analysis of variance (PERMANOVA function) (Anderson, 2001) with 10000 permutations. We used non-metric multidimensional scaling (NMDS), Principal Coordinate (PCO) and Principal Coordinate Canonical analysis (CAP) to build a constrained ordination of the OTUs or groups of other taxonomic levels, based on the environmental data. Then the environmental and biological data were compared by means of Spearman's rank correlation coefficient, using the BEST command. Finally, we generated heat maps for community comparison using TreeView3 software (Saldanha, 2004). We evaluated two factors: Lake (levels: LN and LE) and Depth (levels: 0.5, 10, and 20 m) by means of the following equation for PERMANOVA Routine:

(1) $$\begin{array}{r} \text{y}=\text{u}+{\text{A}}_{\text{lake}}+{\text{B}}_{\text{depth}}+\text{e} \end{array}$$

Where u = mean; A _lake_ = effect of lake; B _depth_ = effect of depth; e = error.

## Results

### Updated climate and hydrological information

**Figure 2:**
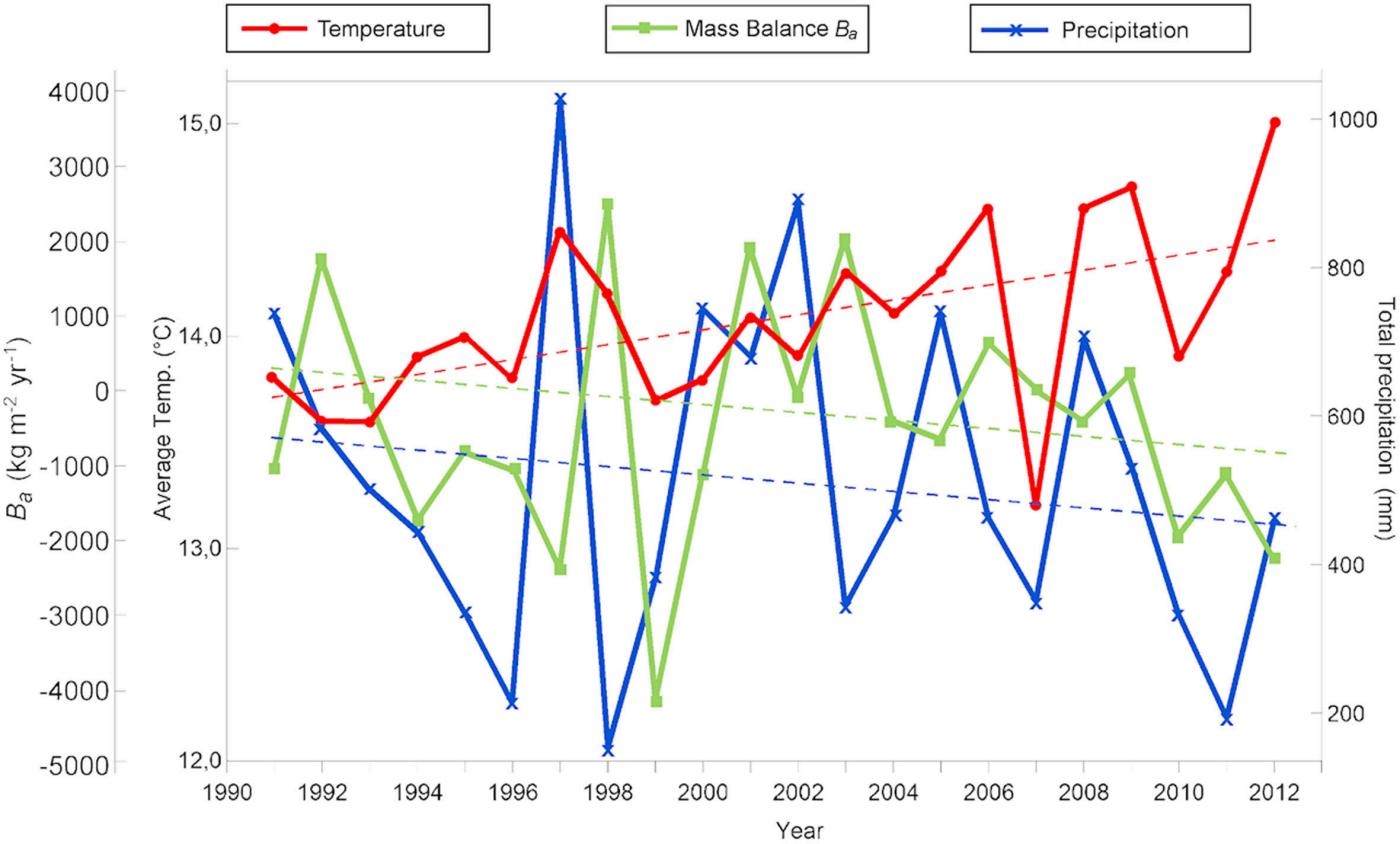
Mean temperature, total precipitation in the zone and mass balance of Echaurren Glacial per year between 1991 and 2012 (Source: Dirección Meteorológica de Chile, 1991–2012; World Glacier Monitoring Service (WGMS), 2015). Dotted lines showed linear regressions.

We collected historical data on precipitation and annual mean temperature from the meteorological station at San José de Maipo, located 20 km east of LN (33° 36′ S−70° 21′ W and 928 masl) and on the streamflow from the Maipo River and the Eucharren Glacier (DGA, http://www.dga.cl/servicioshidrometeorologicos/Paginas/default.aspx). Available climate data collected between 1991 and 2012 show that precipitation has been decreasing (~560–460 mm) and temperature has been increasing (13.8–14.5°C) in the study area following the observed tendency in recent decades (Dirección Meteorológica de Chile, 1991–2012). The increase in temperature has led to the increased melting of Echaurren glacier which showed a negative mass-balance on average from 1993 to 2012 (Figure 2) (Comitato Ev-K2-CNR, 2012; Pellicciotti et al., 2014; Mernild et al., 2015; WGMS et al., 2015).

Hydrological data from DGA was analyzed from 1975 to 2017, for the following parameters: (1) detrital content of Maipo River at Manzano because this river is the final receptor of the runoff waters that come from the LN and LE drainage systems, and (2) the streamflow of Echaurren Glacier. Both records were analyzed with daily resolution. This data allowed us to infer the influence of climatic changes in the response of water resources in the study area since the glacier dynamics finally controls the streamflow existing in the drainage system as snow-melting. The effect of the rainfall was also evaluated.

The detrital content of the Maipo River reflects the amount of sediments that are associated with the erosion of the catchment basin. This parameter is also strongly correlated to the streamflow of the river. From 1975 to 1994, the Maipo River had a daily average of 11,570 tons of eroded sediments, a total sum of 57,296,159 tons, and a daily maximum of 422,023 tons. Nevertheless, from 1994 and 2017, the hydrological dynamics changed, since the Maipo River showed a daily average of only 4,855 tons of eroded sediment, a total sum of 31,971,045 tons, and a daily maximum of 128,557 tons. These values show evidence of the influence of climatic changes on the hydrological characteristics of the study area, and indicate a decreasing trend in the streamflow of the Maipo River during the last 20 years. The increasing trend in temperature and the decreasing extension of glaciers are correlated phenomena with the decreasing streamflow of the Maipo River. The Echaurren Glacier was analyzed for the period 1975–2005. No data was available for the last decade. From 1975 to 1994, the streamflow of Echaurren Glacier associated with snow-melting had a daily average of 0.45 m^3^/s and maximum values of 1.21 m^3^/s. As with the Maipo River, the streamflow record for Echaurren Glacier also decreased from 1994 to 2005, since the streamflow had a daily average of only 0.25 m^3^/s and a maximum daily value of 0.63 m^3^/s.

### Limnological data

**Figure 3:**
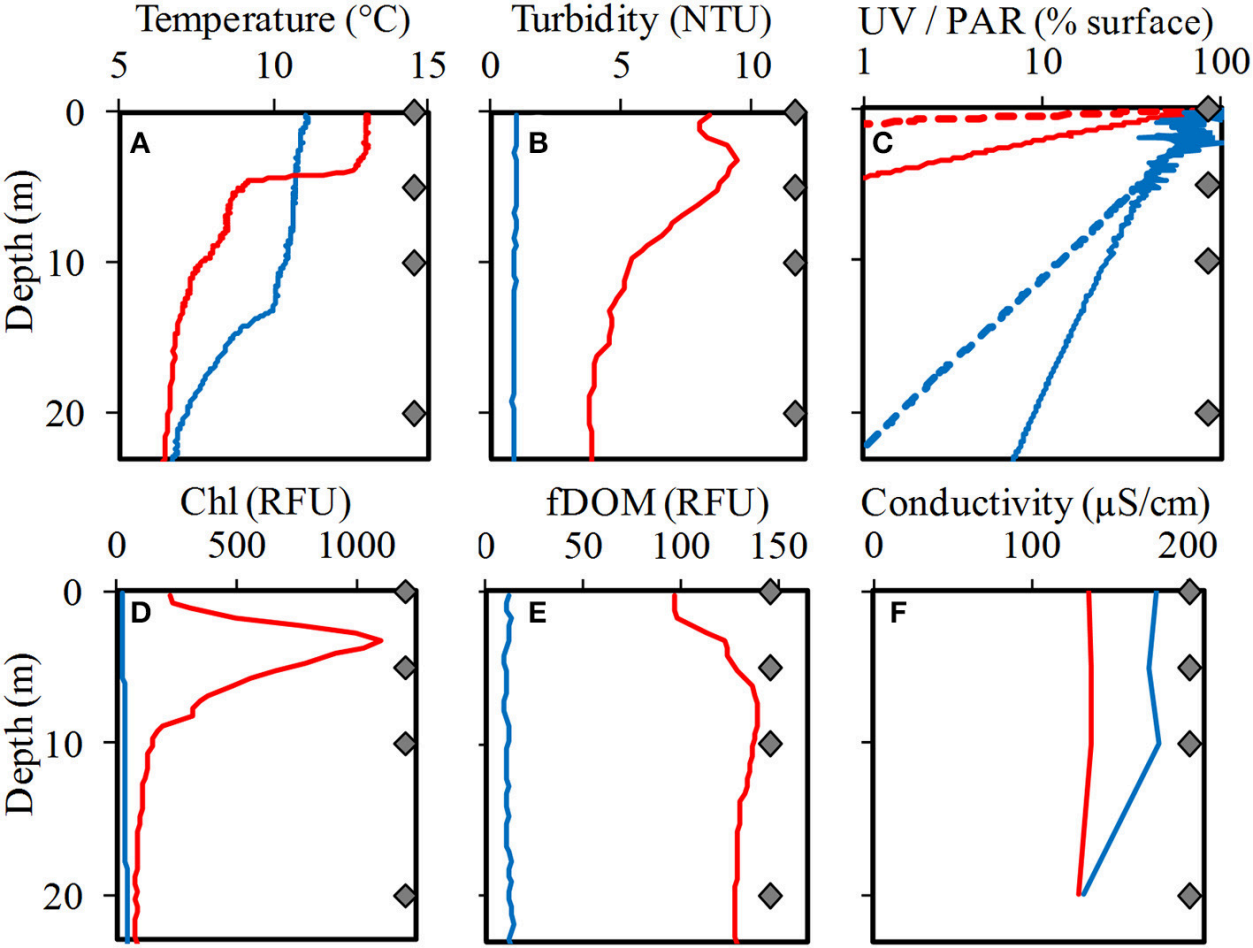
Profiles of temperature **(A)**, turbidity **(B)**, light (UV and PAR) **(C)**, chlorophyll-*a* fluorescence **(D)**, fluorescent dissolved organic matter (fDOM) **(E)**, and conductivity **(F)** in Laguna Negra (blue lines) and Lo Encañado (red lines). For **(C)**, UV profiles are dashed, and PAR profiles are solid lines. NTU is nephelometric turbidity units and RFU is relative fluorescent units. The four gray diamonds on each graph represent the sampling depths.

The chemical and physical characteristics of the water samples corresponding to a single depth profile from both lakes were analyzed and were summarized in Tables 1, 2. The light attenuation measured at 305, 320, 380 nm and PAR (Figure 3) showed a high correlation coefficient >95% among them, and were grouped as a unique variable (we used the 380 nm values as representative) named “Light penetration” for the comparative statistical analyses. The analysis performed using PERMANOVA revealed significant differences in the chemical and physical variables between the two lakes (*p* < 0.05) (Supplementary Figure S1). Secchi disk measurement was 30 m for LN and 10 m for LE and a higher turbidity and a lower water clarity were determined in LE compared to LN (Table 2). The major ion average concentrations (Ca, Na, and Mg) were <2 times higher in LE compared to LN (Table 1). The Chl*a* and the fDOM levels were also higher in LE than in LN (29-55 and 83-727 RFU, respectively−11.8 and 0.4 μgL^−1^) (Rose et al., 2014) on average-; 10-14 and 97-137 RFU, respectively −0.4 and 0.3 mg L^−1^ (Rose et al., 2014). Most of the vertical profiles from LN showed fairly homogeneous physical and chemical variables throughout the water column up to 20 m (Figure 3). The temperature was stable at the surface up to a depth of 13 m and then it gradually decreased (Figure 3). At this time, temperature and specific conductivity were around 9.9°C and 135.9 mS cm^−1^, respectively. On the contrary, LE showed a well-defined vertical stratification at the time of sampling. The temperature was 13.03°C at the surface, it decreased more than 4°C at 5 m, and after that, a gradual decrease was observed. In spite of the conductivity profile which showed only a slight decrease at 20 m in LE, isolated cations analyzed showed an increase concentration from 5 to 10 m depth which followed the turbidity profile (Figure 3). In addition, the turbidity, the fDOM and the Chl*a* showed a peak at 5 m depth. A sharp decrease in the light availability was also evidenced in the water column of LE (Figure 3), while in LN more than 5% of the surface PAR penetrated up to 20 m depth, in LE <1% of the surface radiation was measured even at 5 m depth (Figure 3, Table 2).

**Table 1:** Summary of chemical characteristics of Laguna Negra (LN) and Lo Encañado (LE).

**Sample**	**Ni**	**Ca**	**Si**	**Na**	**K**	**Mg**	**As**	**CI**	**F**	**Nitrate**	**Sulfate**
LN0	0.03	20.1	2.8	3	3.05	1.9	UDL	6.81	0	0	32.46
LN5	0.02	18.2	3.6	3.06	1.3	1.8	UDL	6.81	0.027	0.083	38.90
LN10	0.02	20.6	2.25	2.4	0.95	1.9	UDL	10.21	0.03	0.017	36.93
LN20	0.01	16.1	0.44	2.11	3.21	1.6	UDL	10.21	0	0	36.84
LE0	0.02	23.1	5.35	3.68	1.14	3.5	0.004	10.21	0.02	0	32.5
LE5	0.03	24.3	2.48	4.65	0.96	5.1	0.002	13.61	0.019	0.013	33.93
LE10	0.03	25.7	2.06	4.69	0.97	5.1	UDL	13.61	0.018	0	34.49
LE20	0.03	24.2	UDL	4.25	1.17	4.9	UDL	10.21	0.02	0	33.79

*All concentrations are expressed in ppm*.

*UDL, Under Detection Limits, for Si < 0.38 ppm, for As < 0.002 ppm. The concentration of arsenic, acetate, tartrate, nitrate, fluor, iron, aluminum and zinc, phosphate, formate and acetate were too close to, or below, the detection limit in all the samples of both lakes and were not included in the table*.

**Table 2:** Summary of physical characteristics of Laguna Negra (LN) and Lo Encañado (LE).

**Name**	**pH**	**Eh (mV)**	**Conductivity (uS/cm)**	**Turbidity (NTU^+^)**	fDOM (RFU^*^)	**Chl*a* a fluorescence (RFU)**	**305 nm (% of surface)**	**320 nm (% of surface)**	**380 nm (% of surface)**	**PAR (% of surface)**	**T (°C)**
LN0	7.1	601	137.3	1.03	10.5	29.49	96.89	97.26	97.64	98.35	10.97
LN5	7.3	586	139.0	1.03	12.1	28.91	24.68	33.58	53.19	36.00	10.69
LN10	7.5	577	137.5	1.26	13.4	38.72	4.65	8.74	23.68	18.24	10.03
LN20	7.4	558	130.0	0.94	13.7	55.50	0.14	0.49	4.25	5.23	8.14
LE0	7.2	509	180.0	7.77	97.5	221.73	94.79	95.02	95.30	95.93	13.03
LE5	6.9	504	175.0	8.72	128.4	726.92	0.00	0.00	0.01	0.63	8.97
LE10	7.0	517	182.0	5.32	137.4	143.68	0.00	0.00	0.00	0.02	7.69
LE20	7.4	495	134.0	3.76	127.6	83.49	0.00	0.00	0.00	0.00	6.94

+ *NTU, Nephelometric Turbidity Units*.

* *RFU, Relative Fluorescence Units*.

### Cell count and microbial diversity

The total microbial counts (obtained by means of DAPI) were on the order of magnitude higher (10^5^-10^4^) in LE than in LN (Table 3) and were similar to the observed in oligotrophic lakes (Schiaffino et al., 2016).

**Table 3:** Summary of the diversity indices and cell count for all the studied samples.

**Zone**	**Sample name**	**Total microorganisms (DAPI) Cell/mL**	**Total Species S**	Total Individuals N^*^	**Shannon H' (log_e_)**	**Simpson 1-γ**
Laguna Negra	LN0	5,93E+04	3,288	73.402	3.503	0.872
	LN5	2.01E+04	2,802	98,678	2.937	0.8102
	LN10	4.12E+04	2,256	84,872	3.414	0.8951
	LN20	7.50E+04	2,597	79,458	3.384	0.8751
Lo Encañado	LE0	1.50E+05	3.745	87.721	4.175	0.9414
	LE5	2.19E+05	3,727	82,814	4.386	0.9615
	LE10	1.22E+05	3,569	88,047	4.266	0.9596
	LE20	2.11E+05	3,233	88,646	3.743	0.8996

* *Before normalization*.

**Figure 4:**
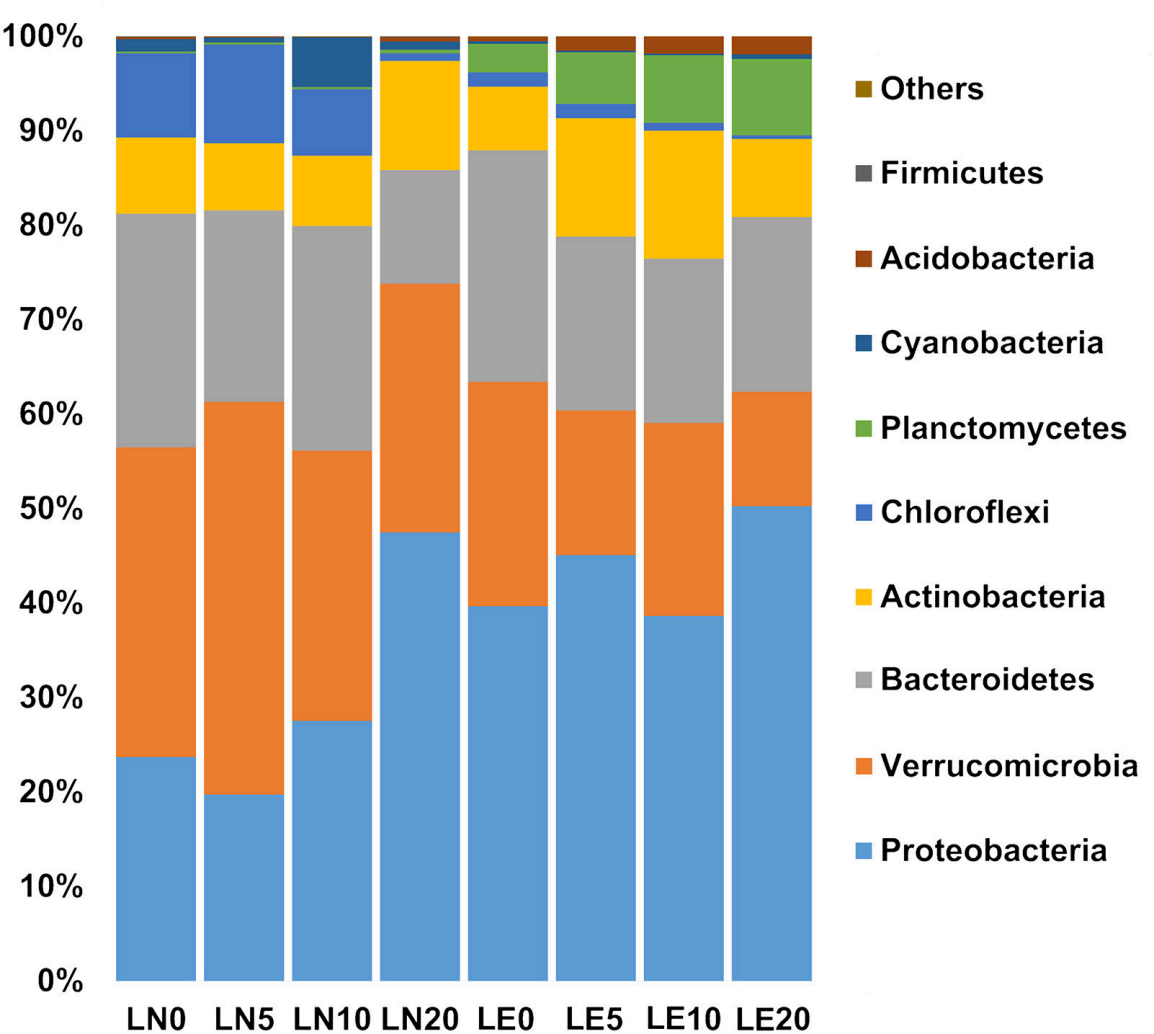
The diversity of the microbial community detected from studied sites at the Phylum level. Stacked column graph representing the relative abundance within the bacteria domain, assessed by analysis of the hypervariable region of bacterial 16S RNA.

The 16S rRNA sequencing analysis resulted in 73,402–98,678 reads (85,455 in average), with data resampled to standardize sequencing effort. This resulted in an average of 3,238 bacterial OTUs (0.03 cut off), which could be classified into 9 representative phyla accounting for 99% of the total sequences in the communities (Figure 4 and Supplementary Figures S2–S9). There were no substantial variations in the number of individuals sequenced among depths or between lakes (PERMANOVA *p* > 0.05) (Table 3). In contrast, univariate statistical indices revealed higher alpha diversity for LE (average H' of 4.1 against 3.3 for LN), while the Simpson index (1-λ = 0.94 vs. 0.86 vs. in LN) was higher in LE (Table 3), which indicates the occurrence of an increased diversity, homogeneity, and number of species compared to LN.

The experimental design considered the analysis of single depth profiles from each lake based on the assumption that those were representatives of the entire lakes because the recorded limnological profiles from both lakes were similar to the ones reported in LN, in 2013, and in Lo Encañado, in 2005 (von Gunten, 2009; Dirección General de Aguas, 2014). Although, there was the possibility that this was not wholly representative of the systems, it allowed us to make microbiological comparisons between similar profiles of the two Andean lakes at the same time.

**Figure 5:**
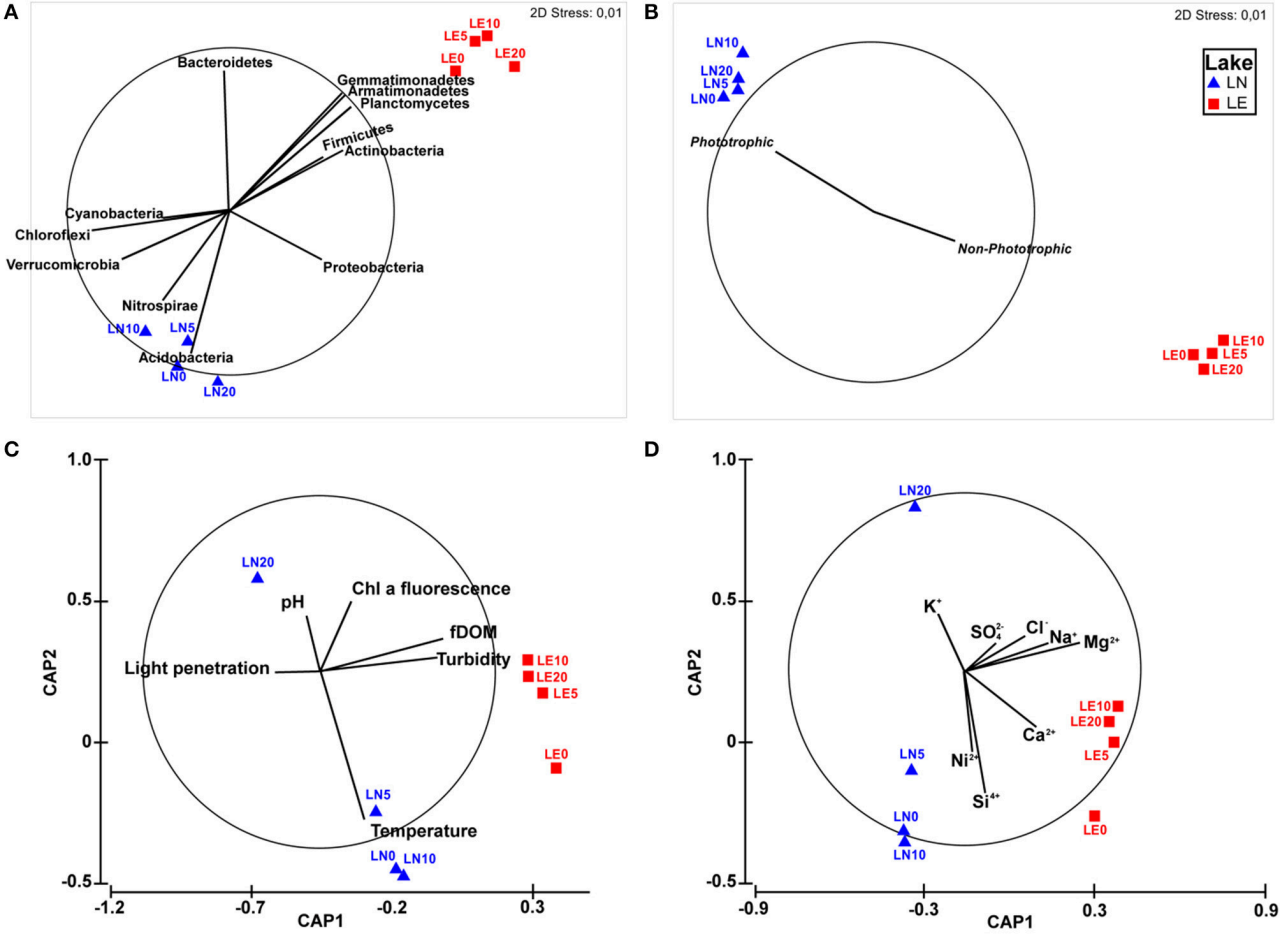
**(A)** Non-metric multidimensional scaling for OTUs, with vectors overlay showing the correlation of principal phyla. **(B)** Non-metric multidimensional scaling for Genus of the principal Phyla, with vectors overlay showing correlation with phototrophy. **(C)** Canonical analysis of principal coordinates (CAP) for OTUs data with vectors overlay showing physical variables. **(D)** Canonical analysis of principal coordinates OTUs with vectors overlay showing chemical variables.

Two well-defined groups were observed in the NMDS analysis but, similar to the physicochemical characteristics, depth was not a discriminating factor for bacterial distribution (Figure 5A), highlighting the fact that differences between the two lakes were far larger than the differences across the depth profiles. Statistical analyses of the results showed that the microbial communities in these profiles significantly correlated with the “Lake” factor (PERMANOVA *p* < 0.05) when Bray Curtis similarities were tested. The five phyla that correlated better with the community distribution (BEST rho = 0.938) were Planctomycetes, Firmicutes, Actinobacteria, Acidobacteria, and Nitrospirae (Figure 5A). Interestingly, the comparison among the taxa belonging to the most representative Phyla of the samples by NMDS showed a strong trend to group by Lake (Figure 5A) due to a clearly higher abundance of phototrophic microorganisms in LN (Figure 5B) than in LE. OTUs related to Cyanobacteria including Chloroplasts, known phototrophic Alpha, Beta and Gammaproteobacteria, Chlorobi and Chloroflexi were considered inside phototrophs to perform this analysis (**Figure 7**). The chemistry—principally by changes in the concentration of Ca, Na, K, Mg and Cl (BEST rho = 0.781)—and physical conditions—turbidity, fDOM, and light penetration (BEST rho = 0.779)—were the variables that better correlated with the microbial composition of both lakes (Figures 5C,D).

**Figure 6:**
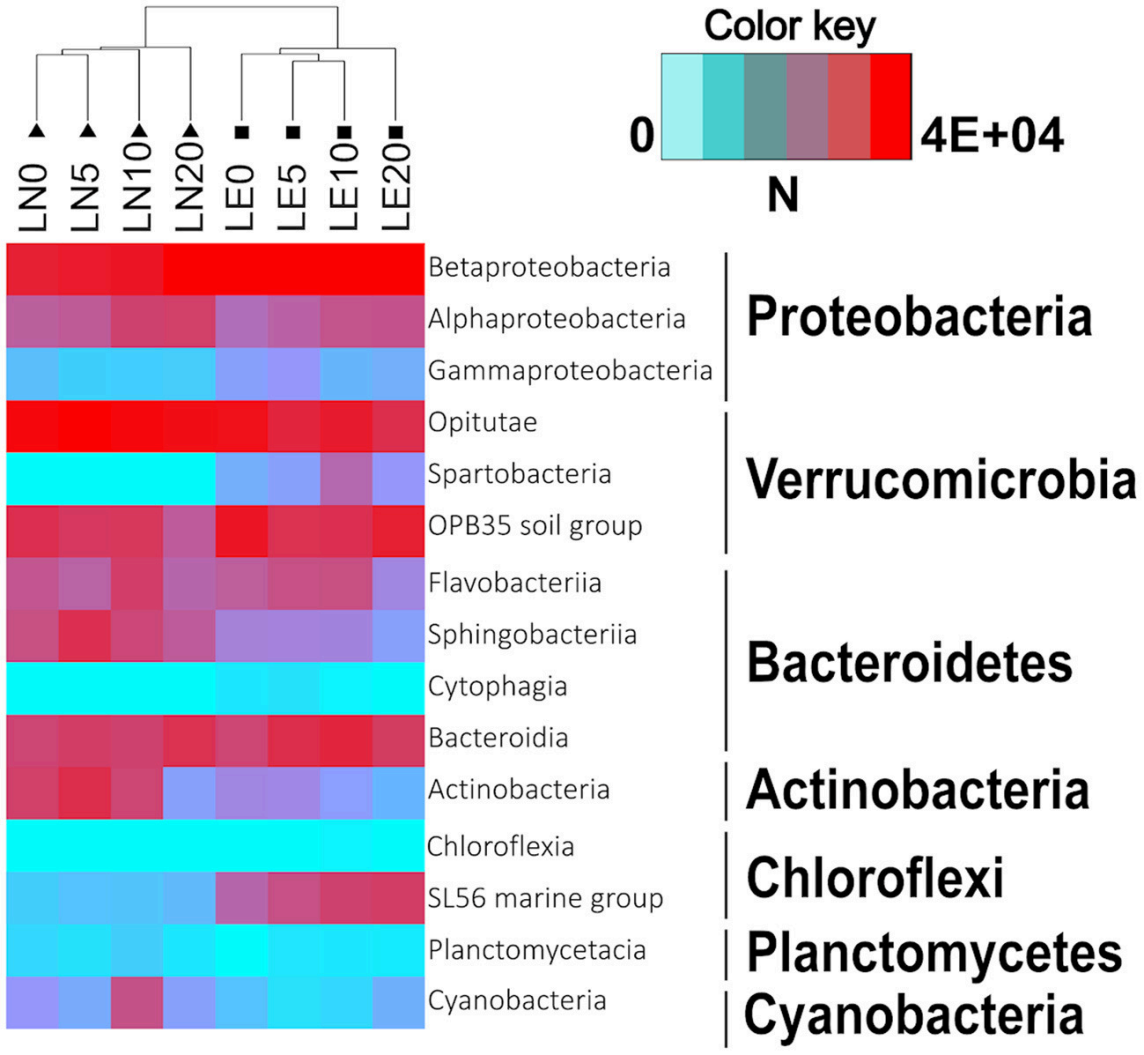
Heatmap showing the abundance of classes of the seven most representative phyla in the samples. Dendrogram shows Bray-Curtis similarities between samples.

**Figure 7:**
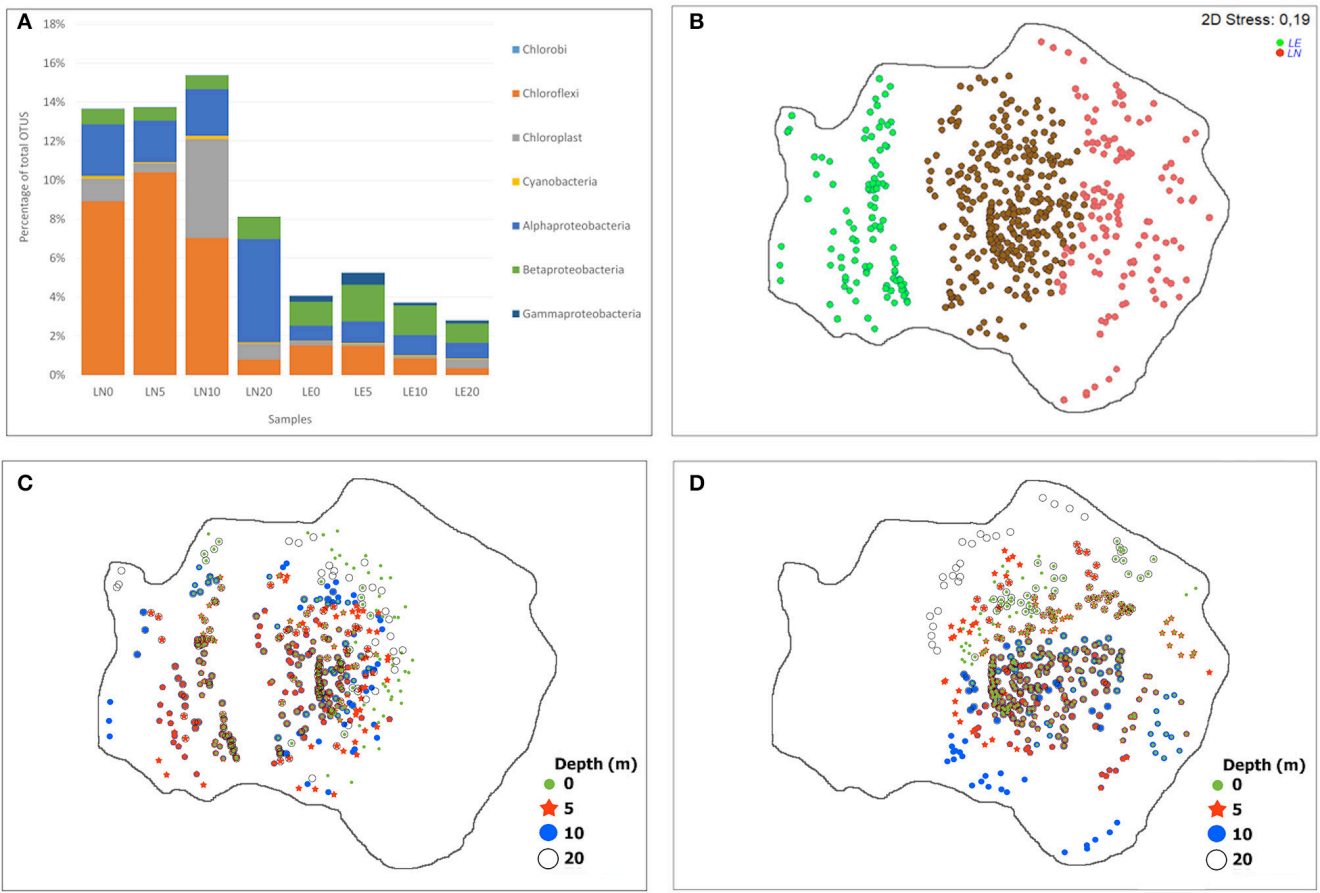
Distribution of the phototrophic phylotypes (OTUs), **(A)** by percentage of each phylum relative to total OTUs, **(B–D)** NMDS showing OTUs distribution between the samples, **(B)** common (brown) and exclusive phylotypes of both lakes (red LN and green LE), **(C)** distribution of the phylotypes present in LE at different depth, **(D)** distribution of the phylotypes present in LN at different depth.

In both lakes, Proteobacteria ranged between 20 and 50% of the lake communities (Figure 4). Noteworthy, the proportions of the two main classes in this phylum were slightly affected by the Lake factor (Figure 6). Betaproteobacteria abundance was 23 and 38% on average in LN and LE, respectively (79 and 88% of the total phylum population, respectively) (Figure 7, Supplementary Figures S2–S9). The OTUs belonging to the genus *Massilia* and *Thiobacillus* were predominant in LN, while OTUs affiliated, at the genus level, to an unclassified Comamonadaceae, were the most abundant in LE (Supplementary Figures S2–S9). Several medium abundance clusters of OTUs, at the genus level, were more abundant in LE and two groups of OTUs belonging to the genus *Chlorochromatium* and LD28 freshwater were mostly found in LE, and one group related to an unclassified Methylophillaceae was only evidenced in LN. In addition, several low abundance OTUs were exclusive from each lake (Supplementary Figures S2–S9).

Alphaproteobacteria accounted for a maximum of 8% in LN and 5% in LE (Figure 4). The cluster of OTUs related to unclassified LD12 fresh water group, at the genus level, was the most abundant member of this group and was more represented in LE, followed in abundance by the clusters related to unclassified BCf3-20 (Rhizobiales), *Sandarakinorhabdus, Novosphingobium* (Sphingomonadaceae), *Roseomonas, Acidiphilium* (Acetobacteraceae), which were more represented in LN (Supplementary Figures S2–S9). A lower number of sequences were affiliated, at the genus level, to *Altererythrobacter, Sphingomonas* and unclassified FukuN57 and were more represented in LN, and to Rhodobacter, Brevundimonas, Rhizobium, unclassified Acetobacteraceae, unclassified alpha cluster, *Methylorosula*, unclassified Bradyrhizobiaceae, *Hyphomicrobium*, unclassified SAR11 clade, unclassified Acetobacteraceae, which were more represented in LE (Supplementary Figures S2–S9). Several clusters with a low number of sequences were observed, some of them more represented in LE and others in LN with a similar distribution (Supplementary Figures S2–S9).

Verrucomicrobia was the second most important group in LE and was predominant in LN up to a depth of 10 m (Figure 4). Together with Proteobacteria, they accounted for more than 50% of the microorganisms present in all the samples (Figure 4). The phylum was almost entirely made up of representatives of the Opitutae family (Figure 6). The OTUs related to the unclassified vadinHA64, at the genus level, were the most abundant in both lakes and the number of sequences was higher in LN than in LE. Other two smaller clusters related to unclassified FukuN18 freshwater group and Opitutus included more sequences from LE than from LN (Supplementary Figures S2–S9).

Chloroflexi accounted, on average, for 7 and 1% in LN and LE, respectively (Figure 4), with more than 96% of OTUs within this group belonging to the Roseiflexus genus (Supplementary Figures S2–S9). The decreased abundance by depth followed the oxygen profile in LN and LE (Dirección General de Aguas, 2014).

No differences were found in the samples with respect to the phylum Bacteroidetes itself (20% average of the bacterial communities in both lakes) (Figure 4), but the distribution of the principal classes showed differences between both lakes (Figure 6, Supplementary Figures S2–S9). Flavobacteria was the most abundant group, representing 9% of the community in LN and 14% in LE, while Cytophagia was present predominantly in LN (7% in LN vs. 1% in LE). The abundance of Sphingobacteria was almost the same in both lakes (5% in LN and vs. 4% in LE). The cluster of OTUs related, at the genus level, to *Flavobacterium*, and an unclassified Cyclobacteriaceae (Flavobacteria class), Candidatus Aquirestis and *Cryomorpha* (Cytophagia class), *Fluviicola* (Saprospiria class), Chitinonophagaeae and *Ferruginibacter* (Chitinophagia class); *Solitalea* (Sphingobacteriia class) were the most represented and they were more abundant in LE (Figure 6, Supplementary Figures S2–S9).

Actinobacteria represented 9 and 10% of the total community in LN and LE, respectively (Figure 4). Genus Hgci_clade (Actinobacteria Genus) accounts for 96% of the Actinobacteria population in the LN and 94% in LE; and as well as the other predominant OTUs, no abundance variations were observed between the samples (Figure 6, Supplementary Figures S2–S9). In addition, there were clusters of OTUs that occurred only in LN (9: *Microbacterium, Phycicoccus, Friedmanniela*, unclassified Acidimicrobiaceae, *Ferritrix, Jatrophihabitans, Frigoribacterium, Frondihabitans, Acidothermus*) and others in LE (5: unclassified Gaiellales, *Gaiella, Corynebacterium, Sporichthya*, and *Alpinimonas*) (Figure 6, Supplementary Figures S2–S9).

Planctomycete and Acidobacteria, on average, were mainly present in LE (6% LE vs. 0.3% in LN and 1.4 vs. 0.2%, respectively) (Figure 6, Supplementary Figures S2–S9). The sequences belonging to the unclassified Planctomycetaceae and CL500-3, at the genus level, were the most abundant of the Planctomycete phylum and were mostly found in LE, while unclassified Holophagaceae marine group, *Acidobacterium* and an unclassified Acidobacteriaceae (Subgroup 1) were the ones with the highest number of sequences belonging to the Acidobacteria phylum. The first one was more represented in LE and the others in LN (Figure 6, Supplementary Figures S2–S9).

Cyanobacteria including chloroplasts represented the 2 and 0.3% in LN and LE, respectively (Figures 4, 6). OTUs belonging to the *Microcystis* and *Synechococcus* genus were the most abundant in LN, while the cluster of OTUs related to the Prochlorococcus genus was more abundant in LE (Figure 6, Supplementary Figures S2–S9). Sequences related to an unclassified ML635J-21 were also found in LN. Cyanobacteria itself was represented by a low abundance in both lakes (0.12 in LN and 0.06 in LE). In addition, Chlorobi was represented by only two sequences found in LN (Figure 6, Supplementary Figures S2–S9).

Archaea were found in both lakes and accounted for <1% of all the microorganisms studied. At 10 m of depth, Euryarchaeota and methanogens dominated in LN, while thermoacidophiles were most abundant in LE (Supplementary Figure S10).

## Discussion

The observed long-term changes in temperature and precipitation were consistent with the results of the hydrological investigations conducted by Migliavacca (Migliavacca et al., 2015) to assess the potential impacts of the climate change until 2100 upon the hydrologic regime of the largely snow-ice melt driven Maipo River basin. Hydrological historical data showed significant changes in the hydrological behavior of the study area during the last 40 years, which are well correlated to climatic changes in northern and central Chile (Center for Climate and Resilience Research (CR)2, 2015; Salas et al., 2016). Overall, in northern and central Chile, higher temperatures trigger greater water loss of glaciers areas by sublimation, as well as lakes and reservoirs by evaporation. As a result, northern and central Chile have experienced a gradual warming since the mid-1970's, and maximum temperatures have been reached rapidly during the last 10 years. In addition, the rainfall records of the area indicated that approximately a quarter of the years from 1940 to 2010 showed precipitation deficits exceeding 30%, i.e., which triggered droughts and significant water deficit (Center for Climate and Resilience Research (CR)2, 2015). The drought periods that were affecting northern and central Chile were also identified in the hydrological record of Eucharren Glacier and the Maipo River at Manzano, by means of a decreasing trend of the streamflow and of the eroded sediments. These drought events also led to the surficial disconnection between LN and LE in 2007, which were initially connected by run-off water from LN to LE.

The chemical composition of lake waters is strongly controlled by the hydrological characteristics in the catchment area since the eroded sediment provides specific major ions to the limnological system (Cumming, 2003; Sarmento et al., 2015). Therefore, the negative mass-balance of Echaurren Glacier (Figure 2) have controlled the chemical composition of LN and LE, since a decrease of streamflow feeding the lakes over the last decades is also triggering a decreasing trend in the inorganic input to the lacustrine systems and their turbidity. In addition, changes in the glacial outflows and the volume of water and concentration of glacially derived substances entering the lakes, in turn, can affect the entire trophic web of the lakes (Yannarell and Triplett, 2005; Slemmons et al., 2013; Sommaruga, 2015).

The observed turbidity, fDOM, light penetration, and major ions levels for both sub-basins (Supplementary Figure S1) were determined by the geological and hydrological differences in the sub-watersheds. The ratio between the volume of the lake and the detrital content related to the meltwater flow was also higher for LN, which helped to increase the difference between both lakes. No evidence of changes in the trophic status of lakes could be observed since our limnological and microbial data could not be compared to previous investigations. Nevertheless, it is expected that variations in runoff water from the Eucharren Glacier may cause fluctuations in nutrients, stratification, and turbidity.

The limnological profiles observed in LN and LE (Figure 3) were similar to those reported in DGA (Dirección General de Aguas, 2014). Therefore, we interpreted that our observations were representative of the entire LN, since the limnological profiles reported by DGA were located along the lake, from the north to the south. The similarities between our profile and all the profiles from DGA (Dirección General de Aguas, 2014) suggested the existence of homogeneous stratification in LN.

Although both lakes receive water from the same glacial source, the sub-watersheds that separate the lakes, as well as the path the glacial water takes before entering the lakes, have led to observable differences between them. These differences are strongly related to the ratio between the sub-watershed areas and the lake areas for LN and LE, which are 4 and 60, respectively, as well as the geology, both triggering variations in the hydrological characteristics and the amount of sediment reaching both lacustrine systems. LN is fed by two different meltwater flows from Echaurren, each of them having at least one pond upstream from LN. These small ponds retain a large proportion of the sediments flowing from the glacier, while LE is fed by meltwater coming directly from the glacier (Figure 1). In addition, the geological units which exist in the catchment of LE are made up of more detrital material compared to the LN catchment (Sernageomin, 2003). This plutonic basement avoids intense erosion process during the run-off, hence providing relatively low detrital content toward the LN. Besides that, the drainage system feeding LE is longer than the one feeding LN and, as a consequence, erosion is providing more amounts of detritus to LE.

The chlorophyll level (determined by Chl*a* fluorescence) and the fDOM were higher in LE than in LN, probably due to the greater abundance of macrophytic algae and the higher degree of turbidity. Upstream wetlands may also be a source of fDOM to LE. The DOM availability could explain the increased cell number evidenced in LE (Table 3) considering that it is known that DOM supports productivity and improves microbial growth in those systems (Hylander et al., 2011; Slemmons et al., 2013). Inputs of glacial meltwater can bring in suspended sediments, thereby reducing light penetration (Hylander et al., 2011; Rose et al., 2014) and decreasing the stress caused by UV radiation (Logue et al., 2012; Slemmons et al., 2013; Sommaruga, 2015) that negatively affects the growth of the heterotrophic flagellates predating over bacteria (Laspoumaderes et al., 2013; Slemmons et al., 2013; Sommaruga and Kandolf, 2014; Sommaruga, 2015). Thus, more favorable conditions may be established for the development of microbial communities as a consequence of glacial inflow (Sommaruga, 2015).

Despite the low concentration of solutes, the sediment samples collected from the water-sediment interface of LE and LN were rich in nutrients (Parro et al., 2016). This was attributed to the colonization of recently deglaciated soils that would increase the influx, the production, and sedimentation of organic matter in the lacustrine system (Parro et al., 2016). The differences in the cell number and indexes from both lakes were consistent with differences found before in the microbial diversity between sediments from both lakes (Parro et al., 2016). In addition, according to the Shannon and Simpson indexes, depth had a slight impact in LE and LN, with an increase in dominance and a decrease in the number of species in the deepest point of the lake in LE and at 5 m depth in LN (Table 3).

The similarity in the most important bacterial groups in both lakes -Proteobacteria, Verrucomicrobia, and Bacteroidetes- suggest that there is a core of relevant functional microorganisms. The phylum Proteobacteria is represented by a large number of Gram-Negative, heterotrophic, phototrophic and nitrogen-fixing organisms, which are at the base of oligotrophic ecosystems (Alfreider et al., 1996; Zwart et al., 2002) and are widely spread in lake systems all over the world (Glöckner et al., 1999; Newton et al., 2011; Schiaffino et al., 2016; Schmidt et al., 2016). The Betaproteobacteria class accounted for almost 90%, in LE, and 80%, in LN, of the sequences of the major Phylum in the system, and included microorganisms with quimiolitotrophic and phototrophic metabolisms which confirms previous results reporting it as the dominant class in freshwater lake systems (Glöckner et al., 1999; Newton et al., 2011). The OTUs associated to Alphaproteobacteria included mainly phototrophic and microorganisms capable of competing for limited nutrients and able to use recalcitrant compounds, such as humic acids (Hutalle-Schmelzer et al., 2010). They were also associated with nitrogen-fixing processes (Newton et al., 2011). A predominant population of Proteobacteria like those we observed in LN and LE was also reported in Patagonian lakes by means of CARD-FISH analysis using specific probes for Alpha, Beta and Gammaproteobacteria, Actinobacteria and Bacteroidetes and a probe to target most Bacteria including Verrucomicrobia and Planctomycetes (Schiaffino et al., 2016).

Verrucomicrobia were previously found in freshwater lakes (Tebo et al., 2015); however, the relative abundance observed in LN and LE was higher (~30 vs. 5%) than the observed in those investigations (Zwart et al., 2002; Newton et al., 2011). This value may have been underestimated since universal primers were not efficient enough to detect Verrucomicrobia (Bergmann et al., 2011). The differential availability of nutrients could be the reason for the higher Verrucomicrobian population in LN compared to LE considering that the comparison performed recently between eleven freshwater lakes showed that this group of bacteria was significantly over-represented in low-nutrient (relative to high-nutrient) lakes (Schmidt et al., 2016). The other potential cause of the differential abundance could be the absence of taxa enrichment in high-nutrient hypolimnia.

Bacteroidetes was the third most important phylum reaching 20% in average with non-significant differences between both lakes. Bacteroidetes was reported as the phylum with the highest average relative abundance across the different habitats studied (free-living, particle-associated, low-nutrient, high-nutrient, hypolimnion, epilimnion, shared) in the lakes from southwestern Michigan (Schmidt et al., 2016). The microorganisms belonging to this phylum were found in freshwater lakes in the presence of organic particles and are central players within the decomposition of complex polymers (Kirchman, 2002; Waidner and Kirchman, 2008). The phylum Actinobacteria includeed a group of ubiquitous bacteria abundant in fresh water systems (Glöckner et al., 2000; Wu and Hahn, 2006; Perez and Sommaruga, 2011). A higher abundance of Actinobacteria was reported in free-living habitats, where the organic matter was dissolved, than in particle-associated habitats (Schmidt et al., 2016).

Interestingly, by assigning phototrophic function based on the phylogenetic affiliation, at the genus level, we found that there was a higher abundance of phototrophic microorganisms in LN compared to LE, 13 vs. 4% on average, respectively (Figure 7). The more abundant phototrophy was related to the light penetration profiles but it was not related to the Chl*a* level in both lakes. The phototrophic population was composed mainly by anoxygenic phototrophic bacteria (AP) (11 and 4% in LN and LE, respectively) harboring to bacteriochlorophyll (Bchl) (Figure 7). The anaerobic AP (AnAP) was predominant in LN accounting for 7 vs. 1%, while the aerobic AP (AAP) comprised 4 and 3% in LN and LE of total bacteria, respectively. The AAP relative contribution is similar to that observed in the oligotrophic and mesotrophic/dystrophic lakes (Salka et al., 2011; Ferrera et al., 2017; Figure 7). Contributions of AAP from 20% up to more than half of the microscopic enumeration bacterial biomass was reported in other oligotrophic water bodies (Masín M et al., 2008). The capacity to harvest light energy was proposed as an important competitive strategy for freshwater bacterioplankton (Martinez-Garcia et al., 2012). The most abundant OTUs related to phototrophs were shared by the community of both lakes and were more represented in LN with the exception of Gammaproteobacteria OTUs that were more abundant in LE. The Cyanobacteria related OTUs (Figure 7) were represented by Microcystis in LN and by Prochlorococcus in LE (Figure 7, Supplementary Figures S2–S9). Several mid-abundance OTUs, mainly related to Chloroplasts and Betaproteobacteria (e.g., Limnohabitans related OTUs in LE and Polaromonas related OTUs in LN), were only evidenced in one of the lakes as well as a large number of low abundant ones (Figure 7, Supplementary Figures S2–S9).

The difference in nutrient availability and temperature of LN and LE could explain the higher AAPs in LN as was previously reported (Masín M et al., 2008; Rigosi et al., 2014; Ferrera et al., 2017). A strong reciprocal relationship was observed between the fraction of AAPs bacteria and the DOC concentration (Masín M et al., 2008).

Chloroflexi was mostly represented by one OTU (96 and 92% in average of the total phylum for LN and LE, respectively) related to an unclassified *Roseiflexus*, which contributed by itself up to 10% and 2% of the total community, from the surface up to 10 and 5 m depth in LN and LE, respectively (Figures 4, 7 and Supplementary Figures S2–S9). The high level of sequence homology found within the phylogenetic cluster was previously reported in *Roseiflexus* (van der Meer et al., 2005; Klatt et al., 2013; Rodionova et al., 2015) and was explained because it could exist under strong influence of stabilizing selection (Boomer et al., 2002; Klatt et al., 2013; Gaisin et al., 2015). *Roseiflexus* was described as obligate thermophilic, phototrophic and filamentous bacteria without chlorosomes that could explain the decrease in their abundance below 10 m depth (Figure 7, Supplementary Figures S2–S9). Roseiflexus is known to be responsible for the red–orange layer in thermophilic cyanobacterial mats (Boomer et al., 2000, 2002, 2009) and it is able to grow heterotrophically by aerobic respiration, photoheterotrophically (using light to incorporate prereduced organic compounds), and photoautotrophically (using light to fix inorganic carbon) (Hanada et al., 2002; van der Meer et al., 2005; Gupta et al., 2013). Another relevant feature of *Roseiflexus* is its ecological/physiological dependence from cyanobacteria in mats (van der Meer et al., 2005; Klatt et al., 2013; Rodionova et al., 2015). The similar distribution of Roseiflexus-like sequences and two unclassified chloroplasts (2) could be evidenced for this dependency (van der Meer et al., 2005); however, the number of sequences of the Roseiflexus-like and the unclassified chloroplasts OTUs were not similar. The occurrence of Chloroflexi in freshwater lakes was associated to the abundance of the taxa Chloroflexi CL500-11 in the oxygenated hypolimnion in the community of the Crater Lake and Lake Biwa (Urbach et al., 2001; Xing et al., 2009; Okazaki et al., 2013, 2017; Schmidt et al., 2016). In addition, photoautotrophy was described in Chloroflexi taxa with sulfide and hydrogen as electron donors (van der Meer et al., 2005). The higher abundance of Roseiflexus-like sequences in LN and in the upper section of the water column (Figure 6) suggested that it was driven by the substantially higher water clarity in this lake and transparency and subsequently that Roseiflexus-like sequences were associated to photoheterotrophic and/or photoautotrophic metabolisms. The occurrence of volcanic activity in the surrounding area of LN and LE could be a source of inorganic electron donors for Roseiflexus-like microorganisms. Another potential substrate for Roseiflexus-like growth is the H_2_ produced by microbial fermentation and/or sulfide produced by sulfate reduction -associated to the sediments (Parro et al., 2016)- which must diffuse to the overlying water (van der Meer et al., 2005). Sulfide concentration of the studied system was not available.

The most abundant phylotypes inside the AAP communities were affiliated to the Alphaproteobacteria in LN (79% contribution to the AAP population) and to the Betaproteobacteria in LE (53% contribution to the AAP population) (Figure 7, Supplementary Figures S2–S10). Previous results showed that the contribution of members from Alphaproteobacteria and Betaproteobacteria of AAP varied largely depending on the lake (Waidner and Kirchman, 2008; Cottrell et al., 2010; Salka et al., 2011; Fauteux et al., 2015). Mongolian lakes and the highest altitude lakes in the Tyrolean Alps (Austria) were dominated by members of the Alphaproteobacteria, while in Pyrenean lakes, freshwater lakes in Germany, North American lakes and in oligotrophic lakes at the Tyrolean Alps (Ferrera et al., 2017), the predominant OTUs were associated to Betaproteobacteria AAP members (Ferrera et al., 2017).

The predominant OTUs related to phototrophic Alphaproteobacteria were *Sandarakinorhabdus, Novosphingobium* (Sphingomonadaceae), *Roseomonas, Acidiphilium* (Acetobacteraceae) and were more abundant in LN (Supplementary Figures S2–S9). Considering that photoheterotrophs were identified by single-cell sequencing inside the LD12 freshwater cluster (Alphaproteobacteria) (Martinez-Garcia et al., 2012), which is among the most abundant OUTs belonging to Alphaproteobacteria in the system, the proportion of AAP Alphaproteobacteria could be increased up to 3.8% on average, if the LD12 related OTUs would correspond to phototrophic microorganisms (Supplementary Figures S2–S9).

Furthermore, OTUs affiliated to *Polynucleobacter, Limnohabitans* and *Polaromonas* genus were predominant in both lakes from Betaproteobacteria (Supplementary Figures S2–S9). *Polynucleobacter* was proposed to be the dominant AAPs in temperate freshwater lakes (Martinez-Garcia et al., 2012). A similar predominant genus was observed in low-salinity lakes (range c. < 0.1–1% salinity) (Caliz and Casamayor, 2014).

Sequences related to the Gammaproteobacteria members were evidenced by a very low number of sequences in LN and contributed in a 12% to the AAP population in LE (Caliz and Casamayor, 2014; Ferrera et al., 2017; Figure 7, Supplementary Figures S2–S10), similar to the situation observed in freshwater environments and opposed to the marine environments.

The major abundance of *Microcystis* in LN corresponded to the depth with an 18% availability of PAR radiation (Figure 7, Supplementary Figures S2–S10). *Microcystis* was characterized by its efficient buoyancy regulation and by the sustained presence of zeaxanthin, which contributes to concentrate the population in the illuminated zone and to dissipate excess excitation energy (Reynolds, 2006). Temperature was reported to be the most important driver of *Microcystis* aeruginosa, while the *Prochlorococcus* abundance followed the Chl *a* profile in LE. *Prochlorococcus* predominance in LE should be explained based on its capacity to efficiently harvest very low levels of light energy (García-Fernández et al., 2004) due to the occurrence of a higher turbidity compared to LN.

The presence of Euryarchaeota and Crenarchaeota reflected findings in previous studies in which these microorganisms were described as ubiquitous in freshwater systems (Keough et al., 2003; Schleper et al., 2005; Chaban et al., 2006; Schiaffino et al., 2016). They are capable of competing for trace nutrients to survive, which enables them to thrive in oligotrophic environments (Lee et al., 1999). Marine Group I, Candidatus Nitrosocaldus and Nitrososphaerales are classified as nitrifying archaea (Stahl and De la Torre, 2012); Methanococci, Methanobacteria, and Methanomicrobia, as methanogens (Thauer et al., 2008); and Thermoplasmata, Thermoprotei, Cenarchaeales, Thermococci and soil Crenarchaeota, as thermoacidophiles (Zaparty and Siebers, 2011; Supplementary Figures S11).

## Conclusions

The limnological signatures of Laguna Negra and Lo Encañado, two oligotrophic Andean lakes which receive water from Eucharren Glacier and are exposed to the same climatic scenario, were driven by the characteristics of the corresponding sub-watersheds.

The abundance of phototrophic bacteria is a significant metabolic difference between the microbial communities of the lakes which is not correlated to the Chl*a* concentration. This feature is due to the predominance of anoxygenic phototrophy which is mainly represented by Chloroflexi, Alpha and Betaproteobacteria in the ecosystem. The best explanatory variables for the differential community composition/function are the nutrient levels (concentration of Ca, Na, K, Mg, and fDOM) and the light availability (turbidity and light penetration) in both lakes. In turn, these variables finally depend on the hydrological characteristics of the respective watersheds.

The transparency, the Chl*a* and fDOM levels, the total number of cells as well as the fraction of the Anoxygenic Phototrophs inside the total prokaryotic community reveal the different status of both lakes.

The abundance of a differential phototrophic bacterioplankton suggests that the ability to utilize solar energy, organic and inorganic substrates is a key function in this oligotrophic mountain lakes from a volcanic geological setting.

A deeper description of the abundance and the role of microbial phototrophy in these lakes is required considering that there is a high abundance of unclassified microorganisms and that we have assumed phototrophy based on the taxonomic affiliation of the 16S gene. The analysis of marker genes of anoxygenic phototrophy and the determination of Bchl*a* levels are needed to better describe the role of anoxygenic phototrophy in those lakes.

## Author contributions

NC, VP, and CD conceived the study; AE-V, AS, YB, and LR performed the sampling; AE-V, OE, and AS extract the DNA for sequencing; AE, MG, and CD performed the bioinformatic analysis; AE-V, GC, and JL performed the description of the geological setting; OE performed the geochemical analysis; KR was in charge of getting and analyzing the radiation information; AE-V, CD, JL, VP, and MM-P drafted and reviewed the manuscript. All authors read and approved the final manuscript.

### Conflict of interest statement

The authors declare that the research was conducted in the absence of any commercial or financial relationships that could be construed as a potential conflict of interest.

## References

[B1] Aceituno P. (1988). On the Functioning of the Southern Oscillation in the South American Sector. Part I: Surface Climate. Monthly Weather Rev. 116, 505–524. 10.1175/1520-0493(1988)116<0505:OTFOTS>2.0.CO;2

[B2] Alfreider A. Pernthaler J. Amann R. Sattler B. Glockner F. O. Wille A.. (1996). Community analysis of the bacterial assemblages in the winter cover and pelagic layers of a high mountain lake by *in situ* hybridization. Appl. Environ. Microbiol. 62, 2138–2144. 16535341 10.1128/aem.62.6.2138-2144.1996 PMC1388879

[B3] Anderson M. J. (2001). A new method for non-parametric multivariate analysis of variance. Austral Ecol. 26, 32–46. 10.1111/j.1442-9993.2001.01070.pp.x

[B4] Bennett M. R. Glasser N. F. (2009). Glacial Geology Ice Sheets and Landforms. Chichester, UK: Wiley-Blackwell.

[B5] Bergmann G. T. Bates S. T. Eilers K. G. Lauber C. L. Caporaso J. G. Walters W. A.. (2011). The under-recognized dominance of Verrucomicrobia in soil bacterial communities. Soil Biol. Biochem. 43, 1450–1455. 10.1016/j.soilbio.2011.03.012 22267877 PMC3260529

[B6] Blass A. Bigler C. Grosjean M. Sturm M. (2007). Decadal-scale autumn temperature reconstruction back to AD 1580 inferred from the varved sediments of Lake Silvaplana (southeastern Swiss Alps). Quat. Res. 68, 184–195. 10.1016/j.yqres.2007.05.004

[B7] Boomer S. M. Lodge D. P. Dutton B. E. Pierson B. (2002). Molecular characterization of novel red green nonsulfur bacteria from five distinct hot spring communities in Yellowstone National Park. Appl. Environ. Microbiol. 68, 346–355. 10.1128/AEM.68.1.346-355.2002 11772644 PMC126577

[B8] Boomer S. M. Noll K. L. Geesey G. G. Dutton B. E. (2009). Formation of multilayered photosynthetic biofilms in an alkaline thermal spring in Yellowstone National Park, Wyoming. Appl. Environ. Microbiol. 75, 2464–2475. 10.1128/AEM.01802-08 19218404 PMC2675224

[B9] Boomer S. M. Pierson B. K. Austinhirst R. Castenholz R. W. (2000). Characterization of novel bacteriochlorophyll-a-containing red filaments from alkaline hot springs in Yellowstone National Park. Arch. Microbiol. 174, 152–161. 10.1007/s002030000189 11041345

[B10] Bown F. Rivera A. (2007). Climate changes and recent glacier behaviour in the Chilean Lake District. Glob. Planet. Chang. 59, 79-86. 10.1016/j.gloplacha.2006.11.015

[B11] Cabrera S. Montecino V. (1987). Productividad primaria en ecosistemas limnicos. Arch. Biol. Med. Exp. 20, 105–116.

[B12] Caliz J. Casamayor E. O. (2014). Environmental controls and composition of anoxygenic photoheterotrophs in ultraoligotrophic high- altitude lakes (Central Pyrenees). Environ. Microbiol. Rep. 6, 145–151. 10.1111/1758-2229.12142 24596287

[B13] Caporaso J. G. Bittinger K. Bushman F. D. DeSantis T. Z. Andersen G. L. Knight R. (2010a). PyNAST: a flexible tool for aligning sequences to a template alignment. Bioinformatics 26, 266–267. 10.1093/bioinformatics/btp636 19914921 PMC2804299

[B14] Caporaso J. G. Kuczynski J. Stombaugh J. Bittinger K. Bushman F. D. Costello E. K.. (2010b). QIIME allows analysis of high-throughput community sequencing data. Nat. Meth. 7, 335–336. 10.1038/nmeth.f.303 20383131 PMC3156573

[B15] Carrasco J. F. Casassa G. Quintana J. (2005). Changes of the 0°C isotherm and the equilibrium line altitude in central Chile during the last quarter of the 20th century. Hydrol. Sci. J. 50:948 10.1623/hysj.2005.50.6.933

[B16] Casassa G. Haeberli W. Jones G. Kaser G. Ribstein P. Rivera A. (2007). Current status of Andean glaciers. Glob. Planet. Change 59, 1–9. 10.1016/j.gloplacha.2006.11.013

[B17] Catalan J. Curtis C. J. Kernan M. (2009). Remote European mountain lake ecosystems: regionalisation and ecological status. Freshw. Biol. 54, 2419–2432. 10.1111/j.1365-2427.2009.02326.x

[B18] Center for Climate Resilience Research (CR)2 (2015). Report to the Nation. The 2010-2015 Mega-Drought: A Lesson for the Future. Santiago, Chile: Universidad de Chile Available online at: http://www.cr2.cl/wp-content/uploads/2015/11/Megadrought_report.pdf

[B19] Chaban B. Ng S. Y. Jarrell K. F. (2006). Archaeal habitats — from the extreme to the ordinary. Can. J. Microbiol. 52, 73–116. 10.1139/w05-147 16541146

[B20] Clarke K. R. (1993). Non-parametric multivariate analyses of changes in community structure. Aus. J. Ecol. 18, 117–143. 10.1111/j.1442-9993.1993.tb00438.x

[B21] Clarke K. Somerfield P. Warwick R. (2014). Change in Marine Communities: An Approach to Statistical Analysis and Interpretation. Plymouth: PRIMER-E

[B22] Comitato Ev-K2-CNR (2012). Plan de accion para la conservación de glaciares ante el cambio climático, informe final, in Programa Plan de Acción Para la Conservación de Glaciares ante Cambio Climático. Nieves, Unidad de Glaciología Y Nieves (MOP), Vol. 1 (Santiago: Dirección General De Aguas, Ministerio de Obras Públicas, Gobierno de Chile), 24–41.

[B23] Cottrell M. T. Ras J. Kirchman D. L. (2010). Bacteriochlorophyll and community structure of aerobic anoxygenic phototrophic bacteria in a particle-rich estuary. ISME J. 4, 945–954. 10.1038/ismej.2010.13 20182527

[B24] Cumming B. (2003). Limnology: lake and river ecosystems. Third Edition. By Robert G Wetzel. Q. Rev. Biol. 78, 368–369. 10.1086/380040

[B25] Dirección Meteorológica de Chile (1991–2012). Anuarios Climatológicos 1991-2012. Anual. Santiago: Dirección General de Aeronáutica Civil

[B26] Dirección General de Aguas (2014). Diagnóstico de la Condición trófica de Cuerpos Lacustres Utilizando Nuevas Herramientas Tecnológicas. Santiago: Ministerio de Obras Públicas, Dirección General de Aguas, Departamento de Conservación y Protección de Recursos Hídricos, Centro de Ecología Aplicada Ltda. S.I.T. N° 347.

[B27] Edgar R. C. (2010). Search and clustering orders of magnitude faster than BLAST. Bioinformatics 26, 2460–2461. 10.1093/bioinformatics/btq461 20709691

[B28] Edgar R. C. Haas B. J. Clemente J. C. Quince C. Knight R. (2011). UCHIME improves sensitivity and speed of chimera detection. Bioinformatics 27, 2194–2200. 10.1093/bioinformatics/btr381 21700674 PMC3150044

[B29] Fauteux L. Cottrell M. T. Kirchman D. L. Borrego C. M. Garcia-Chaves M. C. Del Giorgio P. A. (2015). Patterns in Abundance, Cell Size and Pigment Content of Aerobic Anoxygenic Phototrophic Bacteria along Environmental Gradients in Northern Lakes. PLoS ONE 10:e0124035. 10.1371/journal.pone.0124035 25927833 PMC4415779

[B30] Feeley H. B. Davis S. Bruen M. Blacklocke S. Kelly-Quinn M. (2012). The impact of a catastrophic storm event on benthic macroinvertebrate communities in upland headwater streams and potential implications for ecological diversity and assessment of ecological status. J. Limnol. 71, 299–308. 10.4081/jlimnol.2012.e32

[B31] Fernández A. Mark B. G. (2016). Modeling modern glacier response to climate changes along the Andes Cordillera: a multi-scale review. J. Adv. Model. Earth Syst. 8, 467–495. 10.1002/2015MS000482

[B32] Ferrera I. Sarmento H. Priscu J. C. Chiuchiolo A. González J. M. Grossart H. P. (2017). Diversity and Distribution of Freshwater Aerobic Anoxygenic Phototrophic Bacteria across a Wide Latitudinal Gradient. Front. Microbiol. 8:175. 10.3389/fmicb.2017.00175 28275369 PMC5320280

[B33] Gaisin V. A. Kalashnikov A. M. Sukhacheva M. V. Namsaraev Z. B. Barhutova D. D. Gorlenko V. M.. (2015). Filamentous anoxygenic phototrophic bacteria from cyanobacterial mats of Alla hot springs (Barguzin Valley, Russia). Extremophiles 19, 1067–1076. 10.1007/s00792-015-0777-7 26290358

[B34] García-Fernández J. M. de Marsac N. T. Diez J. (2004). Streamlined regulation and gene loss as adaptive mechanisms in Prochlorococcus for optimized nitrogen utilization in oligotrophic environments. Microbiol. Mol. Biol. Rev. 68, 630–638. 10.1128/MMBR.68.4.630-638.2004 15590777 PMC539009

[B35] Glöckner F. O. Fuchs B. M. Amann R. (1999). Bacterioplankton compositions of lakes and oceans: a first comparison based on fluorescence *in situ* hybridization. Appl. Environ. Microbiol. 65, 3721–3726. 10427073 10.1128/aem.65.8.3721-3726.1999 PMC91558

[B36] Glöckner F. O. Zaichikov E. Belkova N. Denissova L. Pernthaler J. Pernthaler A.. (2000). Comparative 16S rRNA analysis of lake bacterioplankton reveals globally distributed phylogenetic clusters including an abundant group of actinobacteria. Appl. Environ. Microbiol. 66, 5053–5065. 10.1128/AEM.66.11.5053-5065.2000 11055963 PMC92419

[B37] Green J. Bohannan B. J. (2006). Spatial scaling of microbial biodiversity. Trends Ecol. Evol. 21, 501–507. 10.1016/j.tree.2006.06.012 16815589

[B38] Gunn J. M. Snucins E. Yan N. D. Arts M. T. (2001). Use of Water Clarity to Monitor the Effects of Climate Change and other Stressors on Oligotrophic Lakes. Environ. Monit. Assess. 67, 69–88. 10.1023/A:1006435721636 11339706

[B39] Gupta R. S. Chander P. George S. (2013). Phylogenetic framework and molecular signatures for the class Chloroflexi and its different clades; proposal for division of the class Chloroflexi class. nov into the suborder Chloroflexineae subord. nov., consisting of the emended family Oscillochloridaceae and the family Chloroflexaceae fam. nov., and the suborder Roseiflexineae subord. nov., containing the family Roseiflexaceae fam. nov. Antonie Van Leeuwenhoek Int. J. General Mol. Microbiol. 103, 99–119. 10.1007/s10482-012-9790-3 22903492

[B40] Hakanson L. Jansson M. (2002). Principles of Lake Sedimentology. New Jersey, NJ: The Blackburn Press.

[B41] Hanada S. Takaichi S. Matsuura K. Nakamura K. (2002). *Roseiflexus castenholzii* gen. nov., sp nov., a thermophilic, filamentous, photosynthetic bacterium that lacks chlorosomes. Int. J. Syst. Evol. Microbiol. 52, 187–193. 10.1099/00207713-52-1-187 11837302

[B42] Härd T. Fan P. Kearns D. R. (1990). A fluorescence study of the binding of HOECHST 33258 and DAPI to halogenated DNAs. Photochem. Photobiol. 51, 77–86. 10.1111/j.1751-1097.1990.tb01686.x 1689498

[B43] Hutalle-Schmelzer K. M. Zwirnmann E. Krüger A. Grossart H. P. (2010). Enrichment and cultivation of pelagic bacteria from a humic lake using phenol and humic matter additions. FEMS Microbiol. Ecol. 72, 58–73. 10.1111/j.1574-6941.2009.00831.x 20459514

[B44] Hylander S. Jephson T. Lebret K. Von Einem J. Fagerberg T. Balseiro E. (2011). Climate-induced input of turbid glacial meltwater affects vertical distribution and community composition of phyto- and zooplankton. J. Plankton Res. 33, 1239–1248. 10.1093/plankt/fbr025

[B45] Julià R. Luque J. A. (2006). Climatic changes vs. catastrophic events in lacustrine systems: a geochemical approach. Quat. Int. 158, 162–171. 10.1016/j.quaint.2006.05.018

[B46] Keough B. P. Schmidt T. M. Hicks R. E. (2003). Archaeal Nucleic Acids in Picoplankton from Great Lakes on Three Continents. Microb. Ecol. 46, 238–248. 10.1007/s00248-003-1003-1 14708748

[B47] Kirchman D. L. (2002). The ecology of Cytophaga-Flavobacteria in aquatic environments. FEMS Microbiol. Ecol. 39, 91–100. 10.1016/S0168-6496(01)00206-9 19709188

[B48] Klatt C. G. Liu Z. F. Ludwig M. Kühl M. Jensen S. I. Bryant D. A.. (2013). Temporal metatranscriptomic patterning in phototrophic Chloroflexi inhabiting a microbial mat in a geothermal spring. Isme J. 7, 1775–1789. 10.1038/ismej.2013.52 23575369 PMC3749495

[B49] Lane D. J. (1991). 16S/23S RRNA Sequencing. Nucleic Acid Techniques in Bacterial Systematics. New York, NY: John Wiley & Sons.

[B50] Laspoumaderes C. Modenutti B. Souza M. S. Bastidas Navarro M. Cuassolo F. Balseiro E. (2013). Glacier melting and stoichiometric implications for lake community structure: zooplankton species distributions across a natural light gradient. Glob. Chang. Biol. 19, 316–326. 10.1111/gcb.12040 23504742

[B51] Lee N. Nielsen P. H. Andreasen K. H. Juretschko S. Nielsen J. L. Schleifer K. H.. (1999). Combination of Fluorescent *In Situ* Hybridization and Microautoradiography—a New Tool for Structure-Function Analyses in Microbial Ecology. Appl. Environ. Microbiol. 65, 1289–1297. 10049895 10.1128/aem.65.3.1289-1297.1999 PMC91176

[B52] Lenchi N. Inceoglu Ö. Kebbouche-Gana S. Gana M. L. Llirós M. Servais P.. (2013). Diversity of microbial communities in production and injection waters of algerian oilfields revealed by 16S rRNA gene amplicon 454 pyrosequencing. PLoS ONE 8:e66588. 10.1371/journal.pone.0066588 23805243 PMC3689743

[B53] Le Quesne C. Acuña C. Boninsegna J. A. Rivera A. Barichivich J. (2009). Long-term glacier variations in the Central Andes of Argentina and Chile, inferred from historical records and tree-ring reconstructed precipitation. Palaeogeogr. Palaeoclimatol. Palaeoecol. 281, 334–344. 10.1016/j.palaeo.2008.01.039

[B54] Logue J. B. Langenheder S. Andersson A. F. Bertilsson S. Drakare S. Lanzén A.. (2012). Freshwater bacterioplankton richness in oligotrophic lakes depends on nutrient availability rather than on species-area relationships. ISME J. 6, 1127–1136. 10.1038/ismej.2011.184 22170419 PMC3358030

[B55] Luque J. A. Julià R. (2002). Lake sediment response to land-use and climate change during the last 1000 years in the oligotrophic Lake Sanabria (northwest of Iberian Peninsula). Sediment. Geol. 148, 343–355. 10.1016/S0037-0738(01)00225-1

[B56] Marion G. M. Fritsen C. H. Eicken H. Payne M. C. (2003). The Search for Life on Europa: limiting Environmental Factors, Potential Habitats, and Earth Analogues. Astrobiology 3, 785–811. 10.1089/153110703322736105 14987483

[B57] Martinez-Garcia M. Swan B. K. Poulton N. J. Gomez M. L. Masland D. Sieracki M. E.. (2012). High-throughput single-cell sequencing identifies photoheterotrophs and chemoautotrophs in freshwater bacterioplankton. Isme J. 6, 113–123. 10.1038/ismej.2011.84 21716306 PMC3246240

[B58] Masín M M. Nedoma J. Pechar L. Koblízek M. (2008). Distribution of aerobic anoxygenic phototrophs in temperate freshwater systems. Environ. Microbiol. 10, 1988–1996. 10.1111/j.1462-2920.2008.01615.x 18430010

[B59] McKay N. P. Kaufman D. S. Michelutti N. (2008). Biogenic silica concentration as a high-resolution, quantitative temperature proxy at Hallet Lake, south-central Alaska. Geophys. Res. Lett. 35, 1–6. 10.1029/2007GL032876

[B60] Mernild S. H. Beckerman A. P. Yde J. C. Hanna E. Malmros J. K. Wilson R. (2015). Mass loss and imbalance of glaciers along the Andes Cordillera to the sub-Antarctic islands. Glob. Planet. Change 133, 109–119. 10.1016/j.gloplacha.2015.08.009

[B61] Migliavacca F. Confortola G. Soncini A. Senese A. Diolaiuti G. A. Smiraglia C. (2015). Hydrology and potential climate changes in the Río Maipo (Chile). Geogr. Fis. Dinam. Quat. 14, 155–168. 10.4461/GFDQ.2015.38.14

[B62] Møller A. K. Søborg D. A. Al-Soud W. A. Sørensen S. J. Kroer N. (2013). Bacterial community structure in High-Arctic snow and freshwater as revealed by pyrosequencing of 16S rRNA genes and cultivation. Polar Res. 32:17390 10.3402/polar.v32i0.17390

[B63] Muyzer G. de Waal E. C. Uitterlinden A. G. (1993). Profiling of complex microbial populations by denaturing gradient gel electrophoresis analysis of polymerase chain reaction-amplified genes coding for 16S rRNA. Appl. Environ. Microbiol. 59, 695–700. 7683183 10.1128/aem.59.3.695-700.1993 PMC202176

[B64] Newton R. J. Jones S. E. Eiler A. McMahon K. D. Bertilsson S. (2011). A Guide to the Natural History of Freshwater Lake Bacteria. Microbiol. Mol. Biol. Rev. 75, 14–49. 10.1128/MMBR.00028-10 21372319 PMC3063352

[B65] Niño-García J. P. Ruiz-González C. del Giorgio P. A. (2016). Interactions between hydrology and water chemistry shape bacterioplankton biogeography across boreal freshwater networks. ISME J. 10, 1755–1766. 10.1038/ismej.2015.226 26849312 PMC4918434

[B66] Okazaki Y. Fujinaga S. Tanaka A. Kohzu A. Oyagi H. Nakano S. (2017). Ubiquity and quantitative significance of bacterioplankton lineages inhabiting the oxygenated hypolimnion of deep freshwater lakes. Isme J. 11, 2279–2293. 10.1038/ismej.2017.89 28585941 PMC5607371

[B67] Okazaki Y. Hodoki Y. Nakano S. (2013). Seasonal dominance of CL500-11 bacterioplankton (phylum Chloroflexi) in the oxygenated hypolimnion of Lake Biwa, Japan. FEMS Microbiol. Ecol. 83, 82–92. 10.1111/j.1574-6941.2012.01451.x 22809435

[B68] Ondov B. D. Bergman N. H. Phillippy A. M. (2011). Interactive metagenomic visualization in a Web browser. BMC Bioinformatics 12:385. 10.1186/1471-2105-12-385 21961884 PMC3190407

[B69] Parro V. Blanco Y. Puente-Sánchez F. Rivas L. A. Moreno-Paz M. Echeverría A.. (2016). Biomarkers and Metabolic Patterns in the Sediments of Evolving Glacial Lakes as a Proxy for Planetary Lake Exploration. Astrobiology. [Epub ahead of print]. 10.1089/ast.2015.1342 27893284

[B70] Parro V. de Diego-Castilla G. Moreno-Paz M. Blanco Y. Cruz-Gil P. Rodríguez-Manfredi J. A.. (2011). A microbial oasis in the hypersaline atacama subsurface discovered by a life detector chip: implications for the search for life on Mars. Astrobiology 11, 969–996. 10.1089/ast.2011.0654 22149750 PMC3242637

[B71] Pellicciotti F. Ragettli S. Carenzo M. McPhee J. (2014). Changes of glaciers in the Andes of Chile and priorities for future work. Sci. Tot. Environ. 493, 1197–1210. 10.1016/j.scitotenv.2013.10.055 24300481

[B72] Perez M. T. Sommaruga R. (2011). Temporal changes in the dominance of major planktonic bacterial groups in an alpine lake: discrepancy with their contribution to bacterial production. Aquat. Microbial. Ecol. 63, 161–170. 10.3354/ame01505

[B73] Pille T. (2013). Event history of the Santiago area (Chile): the Sedimentological Archive of Lago Lo Encañado. Thesis PhD, Ghent University.

[B74] Pizarro J. Vergara P. M. Cerda S. Briones D. (2016). Cooling and eutrophication of southern Chilean lakes. Sci. Tot. Environ. 541, 683–691. 10.1016/j.scitotenv.2015.09.105 26437345

[B75] Pizarro R. Balocchi F. Vera M. Aguilera A. Morales C. Valdés R. (2013). Influencia del cambio climático en el comportamiento de los caudales máximos en la zona Mediterránea de Chile. Tecnol. Ciencias del Agua 4, 05–19.

[B76] Reynolds C. (2006). Ecology of Ohytoplankton. Cambridge, UK: Cambridge University Press.

[B77] Rice E. W. Baird R. B. Eaton A. D. Clesceri L. S. (2012). Standard Methods for the Examination of Water and Wastewater. U.S.A. American Public Health Association, American Water Works Association, Water Environment Federation.

[B78] Rigosi A. Carey C. C. Ibelings B. W. Brookes J. D. (2014). The interaction between climate warming and eutrophication to promote cyanobacteria is dependent on trophic state and varies among taxa. Limnol. Oceanogr. 59, 99–114. 10.4319/lo.2014.59.1.0099

[B79] Rivera A. Acuña C. Casassa G. Bown F. (2002). Use of remotely sensed and field data to estimate the contribution of Chilean glaciers to eustatic sea-level rise. Annals Glaciol. 34, 367–372. 10.3189/172756402781817734

[B80] Rodionova I. A. Li X. Plymale A. E. Motamedchaboki K. Konopka A. E. Romine M. F.. (2015). Genomic distribution of B-vitamin auxotrophy and uptake transporters in environmental bacteria from the *Chloroflexi phylum*. Environ. Microbiol. Rep. 7, 204–210. 10.1111/1758-2229.12227 25345570

[B81] Rose K. C. Hamilton D. P. Williamson C. E. McBride C. G. Fischer J. M. Olson M. H. (2014). Light attenuation characteristics of glacially-fed lakes. J. Geophys. Res. Biogeosci. 119, 1446–1457. 10.1002/2014JG002674

[B82] Salas I. Herrera C. Luque J. A. Delgado J. Urrutia J. Jordan T. (2016). Recent climatic events controlling the hydrological and the aquifer dynamics at arid areas: The case of Huasco River watershed, northern Chile. Sci. Tot. Environ. 571(Suppl. C), 178–194. 10.1016/j.scitotenv.2016.07.132 27471983

[B83] Saldanha A. J. (2004). Java Treeview - extensible visualization of microarray data. Bioinformatics 20, 3246–3248. 10.1093/bioinformatics/bth349 15180930

[B84] Salka I. Cuperová Z. Mašín M. KoblíŽek M. Grossart H. P. (2011). Rhodoferax-related pufM gene cluster dominates the aerobic anoxygenic phototrophic communities in German freshwater lakes. Environ. Microbiol. 13, 2865–2875. 10.1111/j.1462-2920.2011.02562.x 21895915

[B85] Sarmento H. Casamayor E. O. Auguet J. C. Vila-Costa M. Felip M. Camarero L.. (2015). Microbial food web components, bulk metabolism, and single-cell physiology of piconeuston in surface microlayers of high-altitude lakes. Front. Microbiol. 6:361. 10.3389/fmicb.2015.00361 25999921 PMC4419848

[B86] Saros J. E. Rose K. C. Clow D. W. Stephens V. C. Nurse A. B. Arnett H. A.. (2010). Melting Alpine Glaciers Enrich High-Elevation Lakes with Reactive Nitrogen. Environ. Sci. Technol. 44, 4891–4896. 10.1021/es100147j 20527763

[B87] Schiaffino M. R. Sanchez M. L. Gerea M. Unrein F. Balague V. Gasol J. M. (2016). Distribution patterns of the abundance of major bacterial and archaeal groups in Patagonian lakes. J. Plankton Res. 38, 64–82. 10.1093/plankt/fbv105

[B88] Schleper C. Jurgens G. Jonuscheit M. (2005). Genomic studies of uncultivated archaea. Nat Rev Micro 3, 479–488. 10.1038/nrmicro1159 15931166

[B89] Schmidt M. L. White J. D. Denef V. J. (2016). Phylogenetic conservation of freshwater lake habitat preference varies between abundant bacterioplankton phyla. Environ. Microbiol. 18, 1212–1226. 10.1111/1462-2920.13143 26631909

[B90] Sernageomin (2003). Mapa Geológico de Chile: Versión Digital. Santiago: Servicio Nacional de Geología y Minería, Publicación Geológica Digital.

[B91] Skoog D. A. West D. M. Holler F. J. Crouch S. R. (2014). Fundamentals of Analytical Chemistry, 9th Edn. Belmont, CA: Brooks/Cole Cengage Learning.

[B92] Slemmons K. E. Saros J. E. Simon K. (2013). The influence of glacial meltwater on alpine aquatic ecosystems: a review. Environ. Sci. Process. Impacts 15, 1794–1806. 10.1039/c3em00243h 24056713

[B93] Sommaruga R. (2015). When glaciers and ice sheets melt: consequences for planktonic organisms. J. Plankton Res. 37, 509–518. 10.1093/plankt/fbv027 26869738 PMC4747089

[B94] Sommaruga R. Casamayor E. O. (2009). Bacterial “cosmopolitanism” and importance of local environmental factors for community composition in remote high-altitude lakes. Freshw. Biol. 55, 994–1005. 10.1111/j.1365-2427.2008.02146.x 20543908 PMC2883735

[B95] Sommaruga R. Kandolf G. (2014). Negative consequences of glacial turbidity for the survival of freshwater planktonic heterotrophic flagellates. Sci. Rep. 4:4113. 10.1038/srep04113 24531332 PMC3925964

[B96] Soranno P. A. Spence Cheruvelil K. Webster K. E. Bremigan M. T. Wagner T. Stow C. A. (2010). Using landscape limnology to classify freshwater ecosystems for multi-ecosystem management and conservation. Bioscience 60, 440–454. 10.1525/bio.2010.60.6.8

[B97] Stahl D. A. De la Torre J. R. (2012). Physiology and diversity of ammonia-oxidizing archaea. Annu. Rev. Microbiol. 66, 83–101. 10.1146/annurev-micro-092611-150128 22994489

[B98] Tebo B. M. Davis R. E. Anitori R. P. Connell L. B. Schiffman P. Staudigel H. (2015). Microbial communities in dark oligotrophic volcanic ice cave ecosystems of Mt. Erebus, Antarctica. Front. Microbiol. 6:179. 10.3389/fmicb.2015.00179 25814983 PMC4356161

[B99] Thauer R. K. Kaster A. K. Seedorf H. Buckel W. Hedderich R. (2008). Methanogenic archaea: ecologically relevant differences in energy conservation. Nat. Rev. Microbiol. 6, 579–591. 10.1038/nrmicro1931 18587410

[B100] Urbach E. Vergin K. L. Young L. Morse A. Larson G. L. Giovannoni S. J. (2001). Unusual bacterioplankton community structure in ultra-oligotrophic Crater Lake. Limnol. Oceanogr. 46, 557–572. 10.4319/lo.2001.46.3.0557

[B101] van der Meer M. T. Schouten S. Bateson M. M. Nübel U. Wieland A. Kühl M.. (2005). Diel variations in carbon metabolism by green nonsulfur-like bacteria in alkaline siliceous hot spring microbial mats from Yellowstone National Park. Appl. Environ. Microbiol. 71, 3978–3986. 10.1128/AEM.71.7.3978-3986.2005 16000812 PMC1168979

[B102] van Duin E. H. S. Blom G. Lijklema L. Scholten M. J. M. (1992). Aspects of modelling sediment transport and light conditions in Lake Marken. Hydrobiologia 235/236, 167-176. 10.1007/BF00026209

[B103] Vicuña S. Garreaud R. D. McPhee J. (2010). Climate change impacts on the hydrology of a snowmelt driven basin in semiarid Chile. Clim. Change 105, 469–488. 10.1007/s10584-010-9888-4

[B104] von Gunten L. (2009). High-Resolution, Quantitative Climate Reconstruction Over the Past 1000 Years and Pollution History Derived from Lake Sediments in Central Chile. Thesis. PhD of Science in Climate Sciences, Universität Bern.

[B105] Waidner L. A. Kirchman D. L. (2008). Diversity and distribution of ecotypes of the aerobic anoxygenic phototrophy gene pufM in the Delaware estuary. Appl. Environ. Microbiol. 74, 4012–4021. 10.1128/AEM.02324-07 18469118 PMC2446510

[B106] WGMS ICSU(WDS), IUGG(IACS), UNEP, UNESCO, and WMO (2015). World Glacier Monitoring Service, Global Glacier Change Bulletin No. 2 (2014-2015). Publication based on database version. Zurich, Switzerland: WGMS 10.5904/wgms-fog-2017-10

[B107] Wu Q. L. Hahn M. W. (2006). Differences in structure and dynamics of Polynucleobacter communities in a temperate and a subtropical lake, revealed at three phylogenetic levels. FEMS Microbiol. Ecol. 57, 67–79. 10.1111/j.1574-6941.2006.00105.x 16819951

[B108] Xing P. Hahn M. W. Wu Q. L. (2009). Low Taxon Richness of Bacterioplankton in High-Altitude Lakes of the Eastern Tibetan Plateau, with a Predominance of Bacteroidetes and *Synechococcus* spp. Appl. Environ. Microbiol. 75, 7017–7025. 10.1128/AEM.01544-09 19767472 PMC2786500

[B109] Xu C. (2017). The landslide that dammed Mengda Lake was not triggered by the 1927 Gulang, China, M8 earthquake. J. Paleolimnol. 57, 157–161. 10.1007/s10933-016-9934-y

[B110] Yannarell A. C. Triplett E. W. (2005). Geographic and Environmental Sources of Variation in Lake Bacterial Community Composition. Appl. Environ. Microbiol. 71, 227–239. 10.1128/AEM.71.1.227-239.2005 15640192 PMC544217

[B111] Zaparty M. Siebers B. (2011). Physiology, Metabolism, and Enzymology of Thermoacidophiles, in Extremophiles Handbook, eds Horikoshi K. Antranikian G. Bull A. T. Robb F. T. Stetter K. O. (Tokyo: Springer), 601–639.

[B112] Zwart G. Crump B. C. Agterveld M. Hagen F. Han S. K. (2002). Typical freshwater bacteria: an analysis of available 16S rRNA gene sequences from plankton of lakes and rivers. Aquat. Microbial. Ecol. 28, 141–155. 10.3354/ame028141

