# Supplementary figures and images for: Watershed-Induced Limnological and Microbial Status in Two Oligotrophic Andean Lakes Exposed to the Same Climatic Scenario
Source: Front Microbiol. 2018 Mar 5;9:357. doi: 10.3389/fmicb.2018.00357 (PMC5844981; doi:10.3389/fmicb.2018.00357)

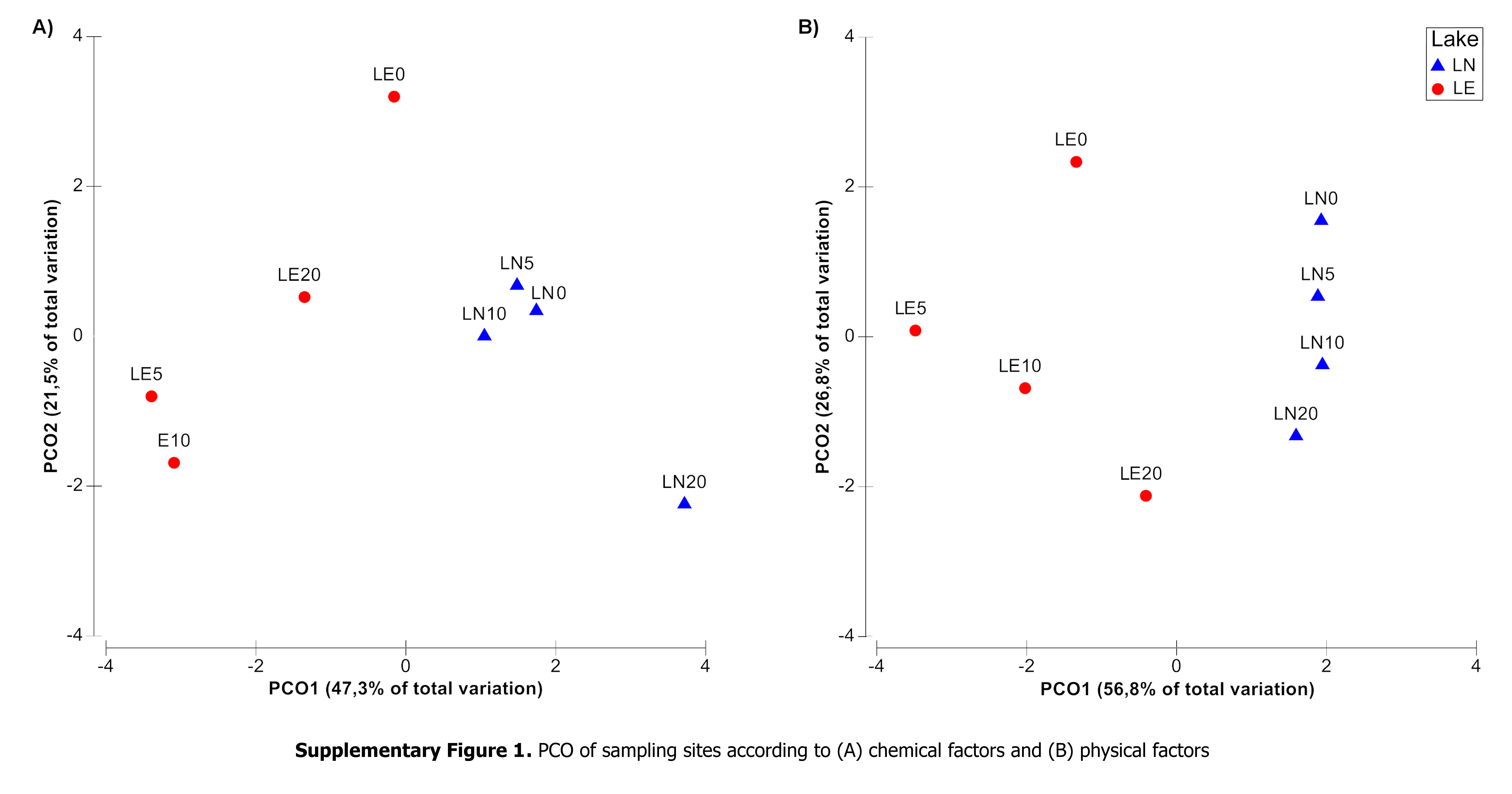

Supplement: Supplementary file 1 [file Image1.JPEG]

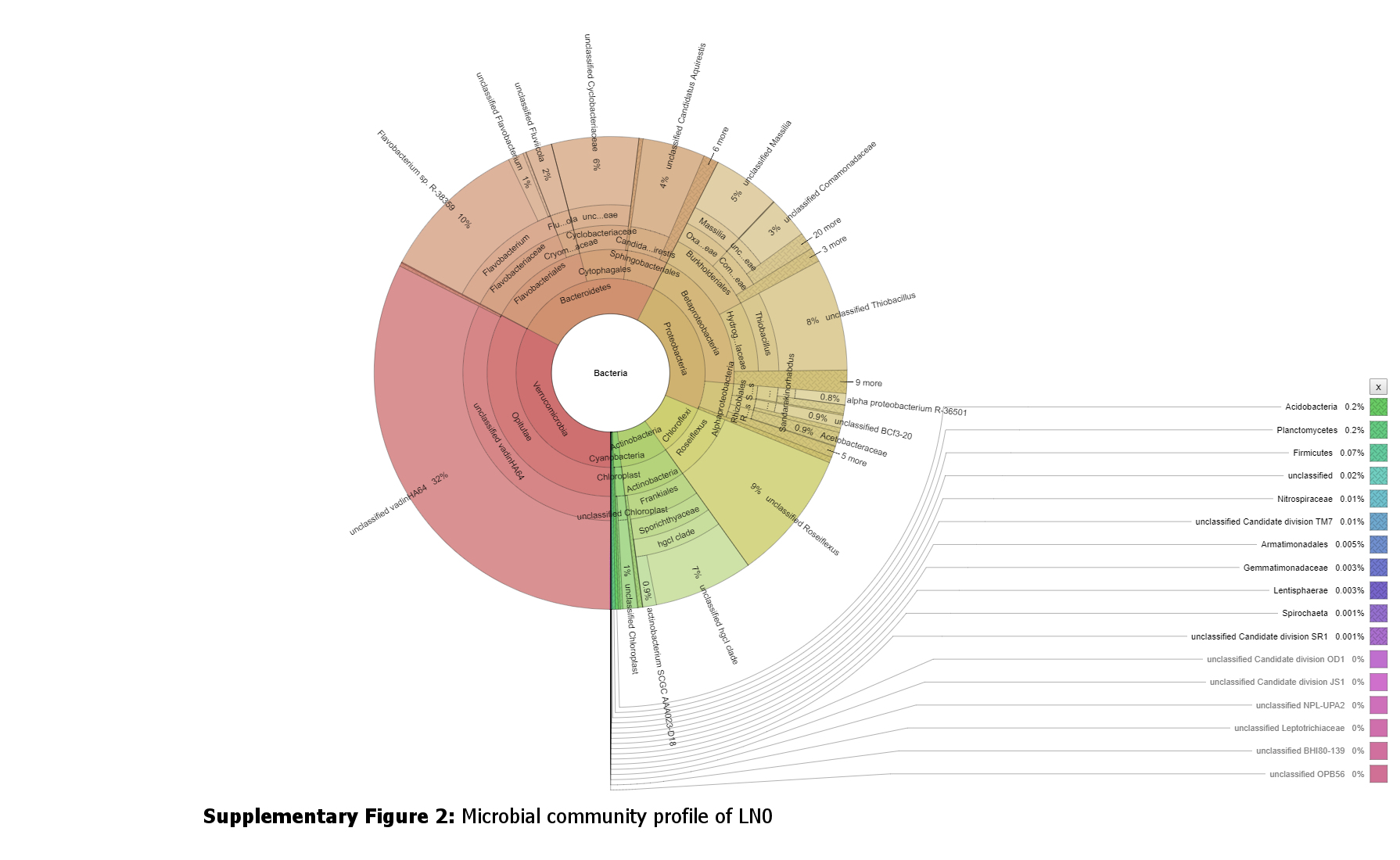

Supplement: Supplementary file 2 [file Image2.JPEG]

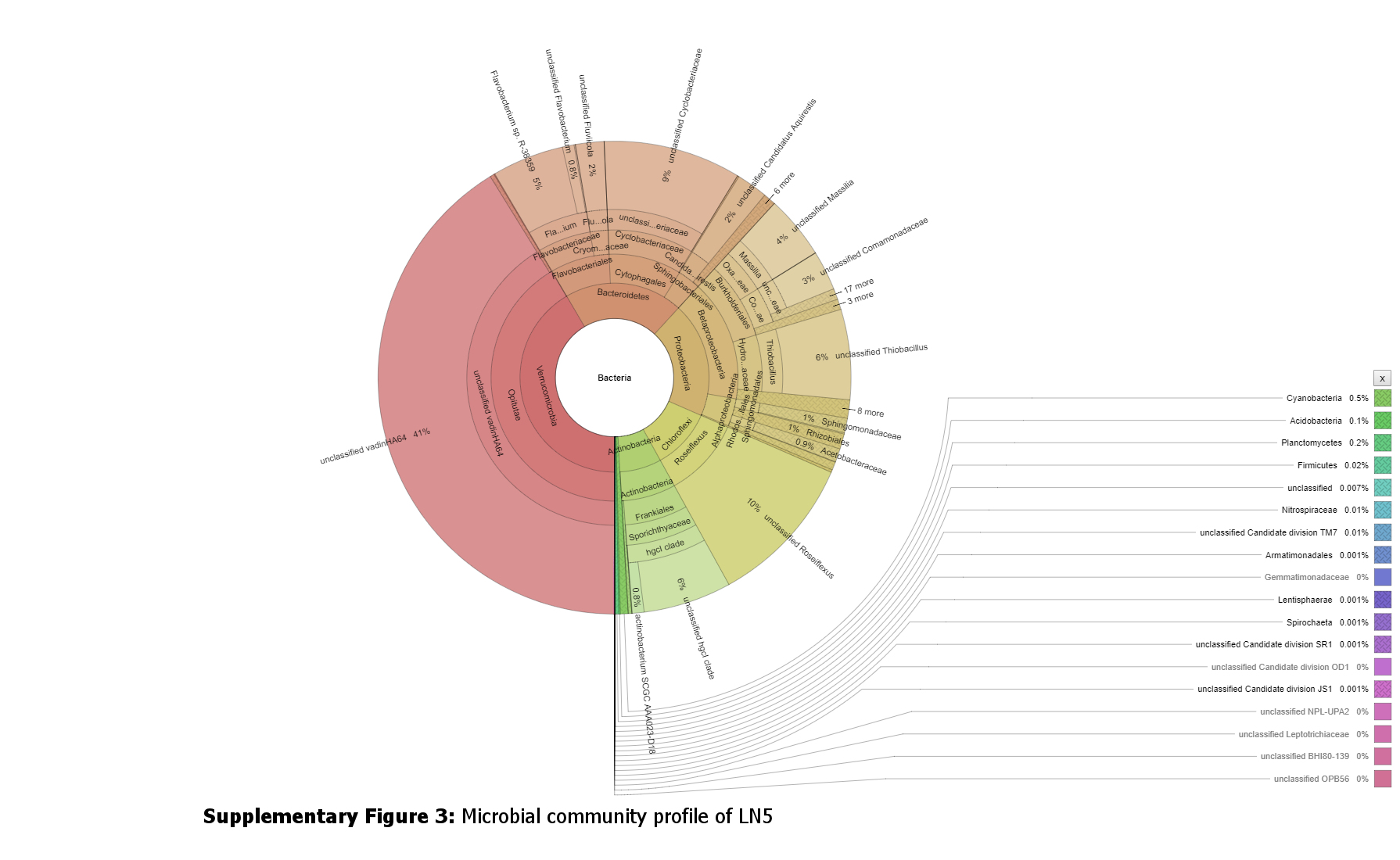

Supplement: Supplementary file 3 [file Image3.JPEG]

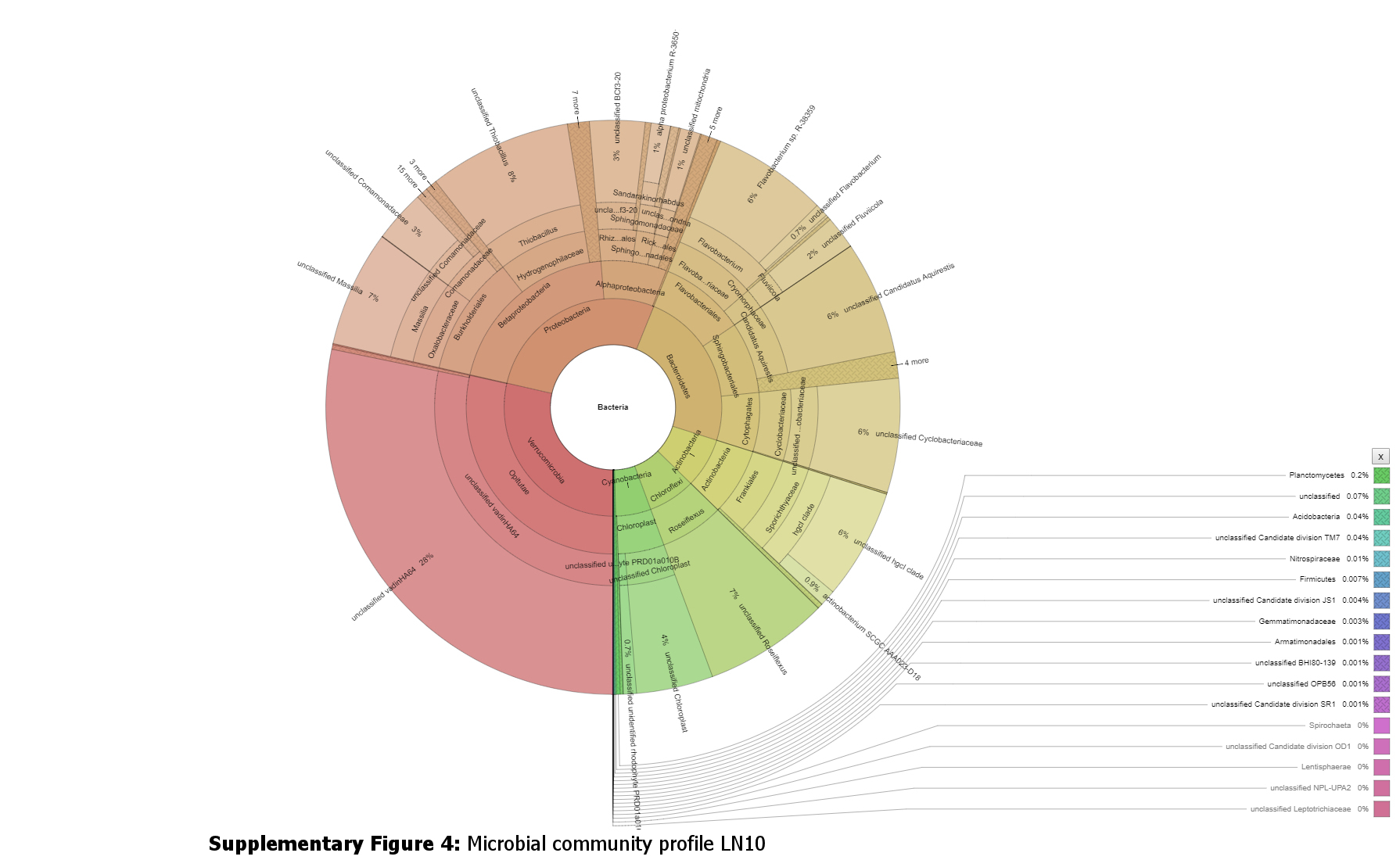

Supplement: Supplementary file 4 [file Image4.JPEG]

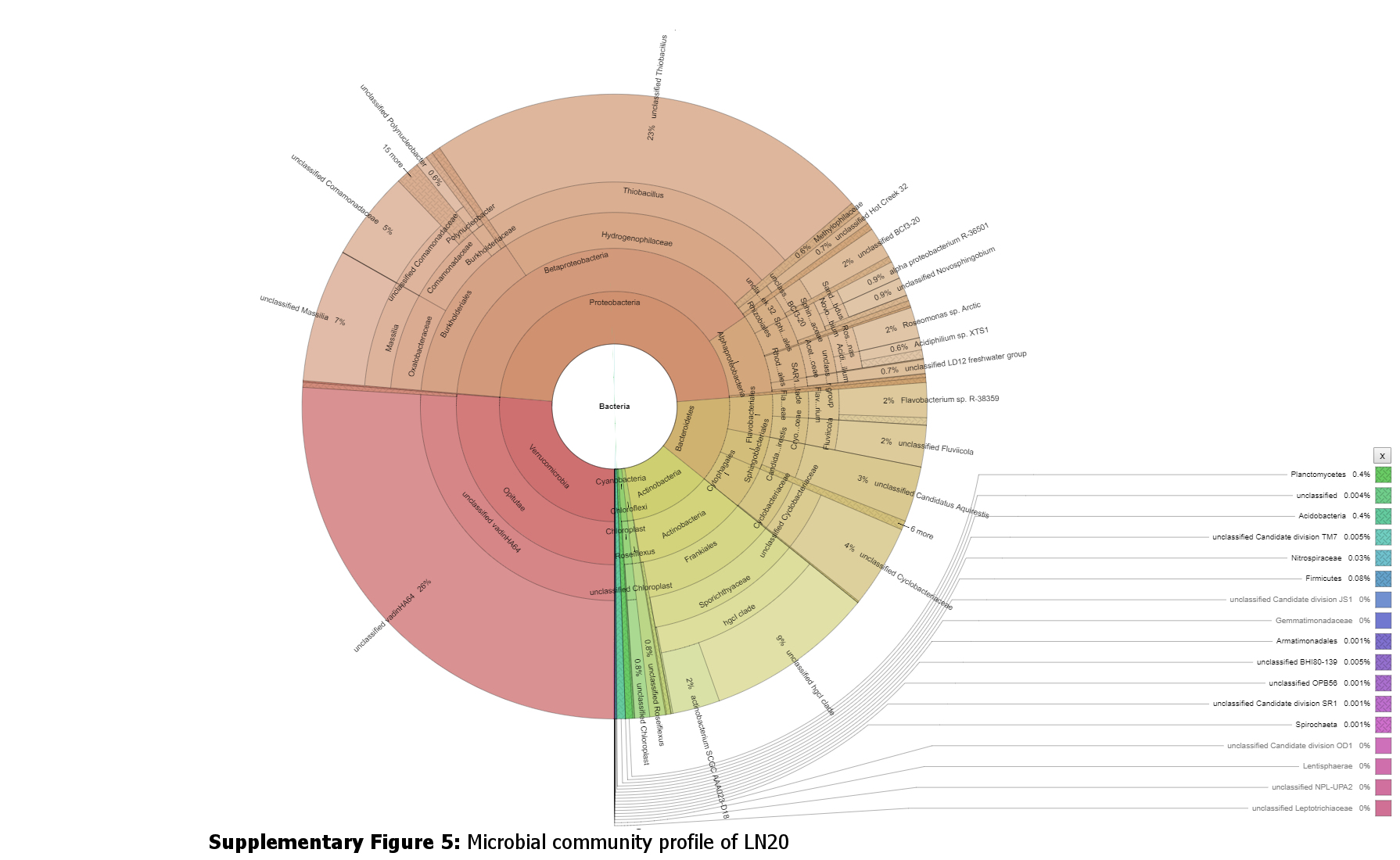

Supplement: Supplementary file 5 [file Image5.JPEG]

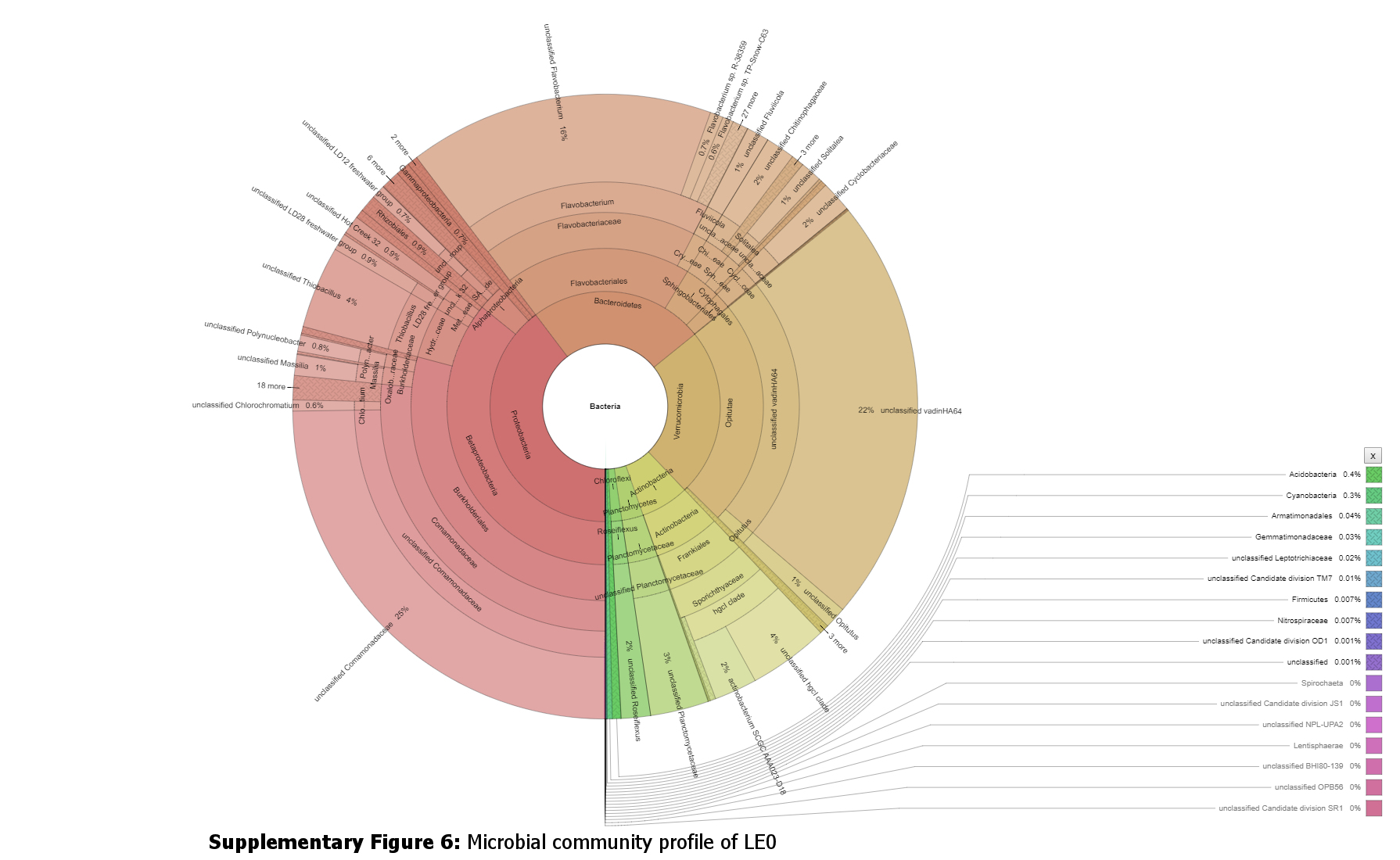

Supplement: Supplementary file 6 [file Image6.JPEG]

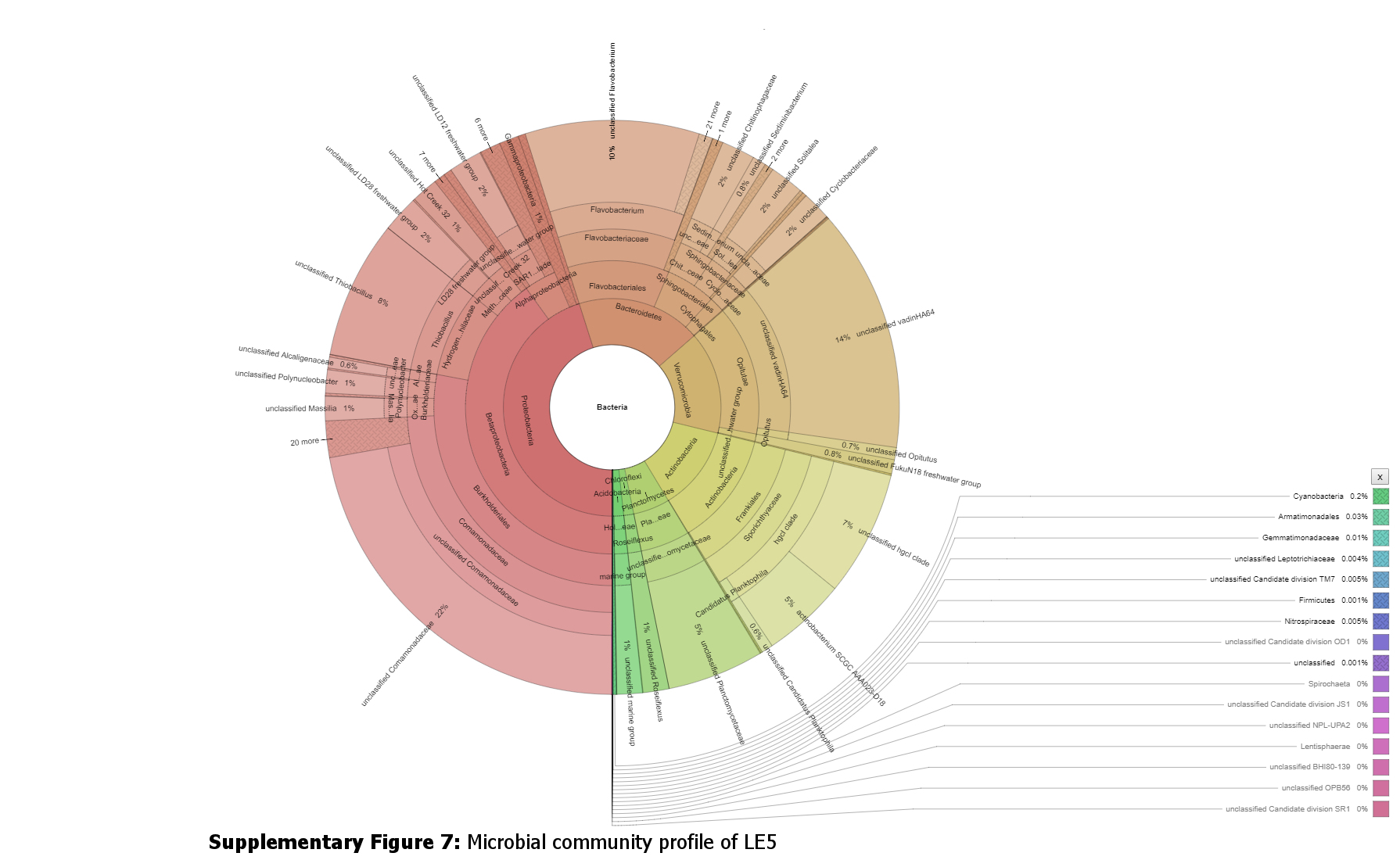

Supplement: Supplementary file 7 [file Image7.JPEG]

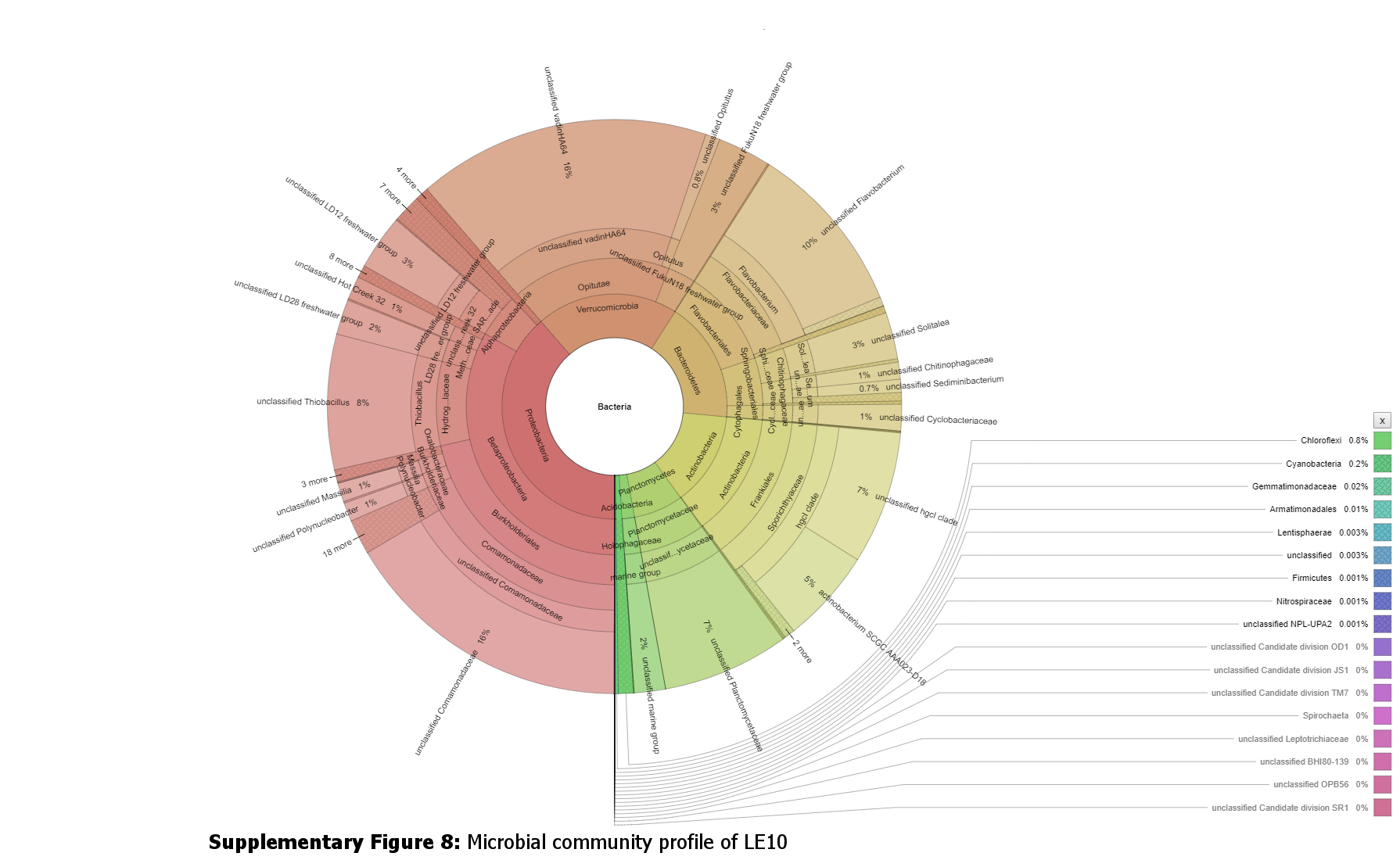

Supplement: Supplementary file 8 [file Image8.JPEG]

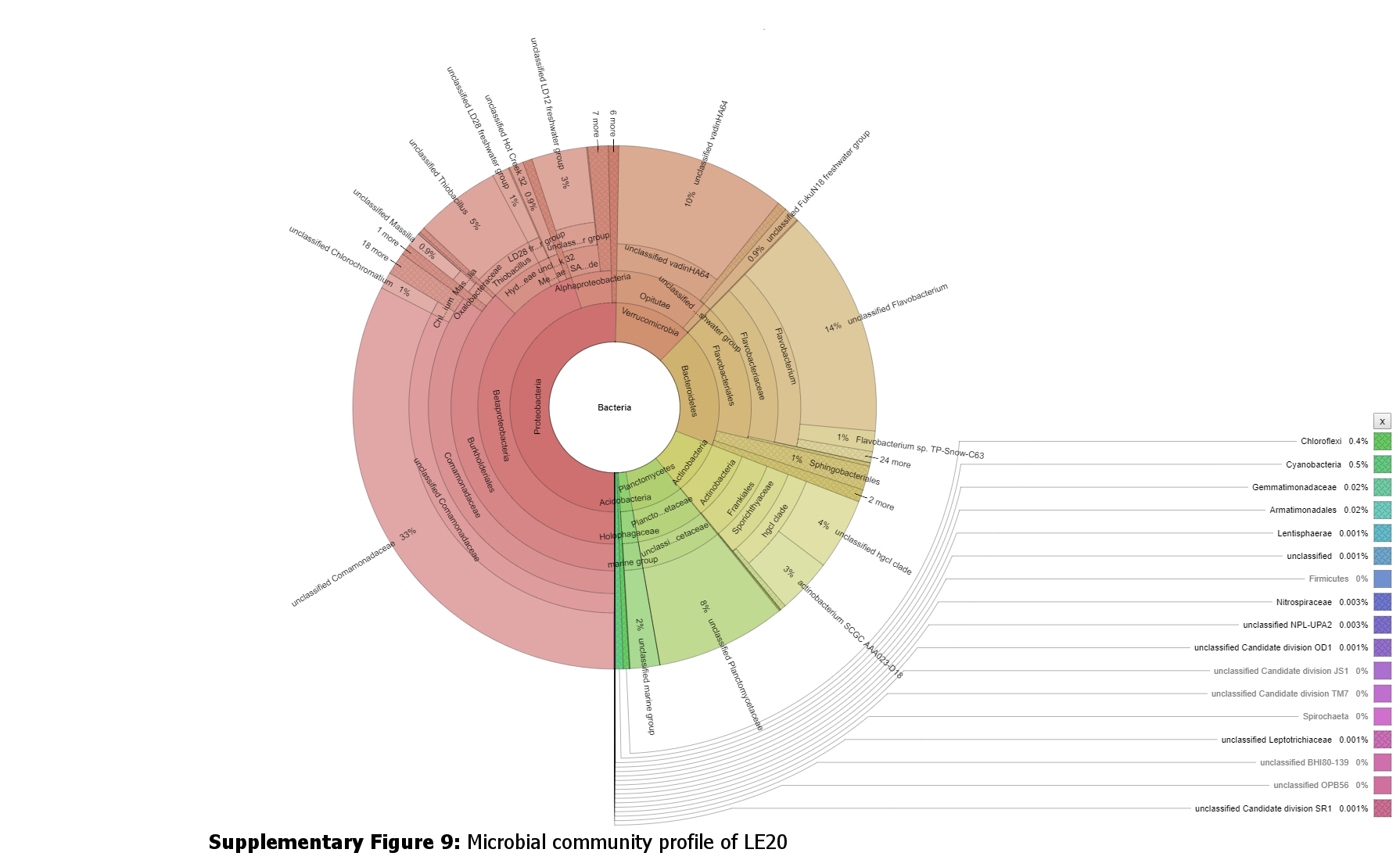

Supplement: Supplementary file 9 [file Image9.JPEG]

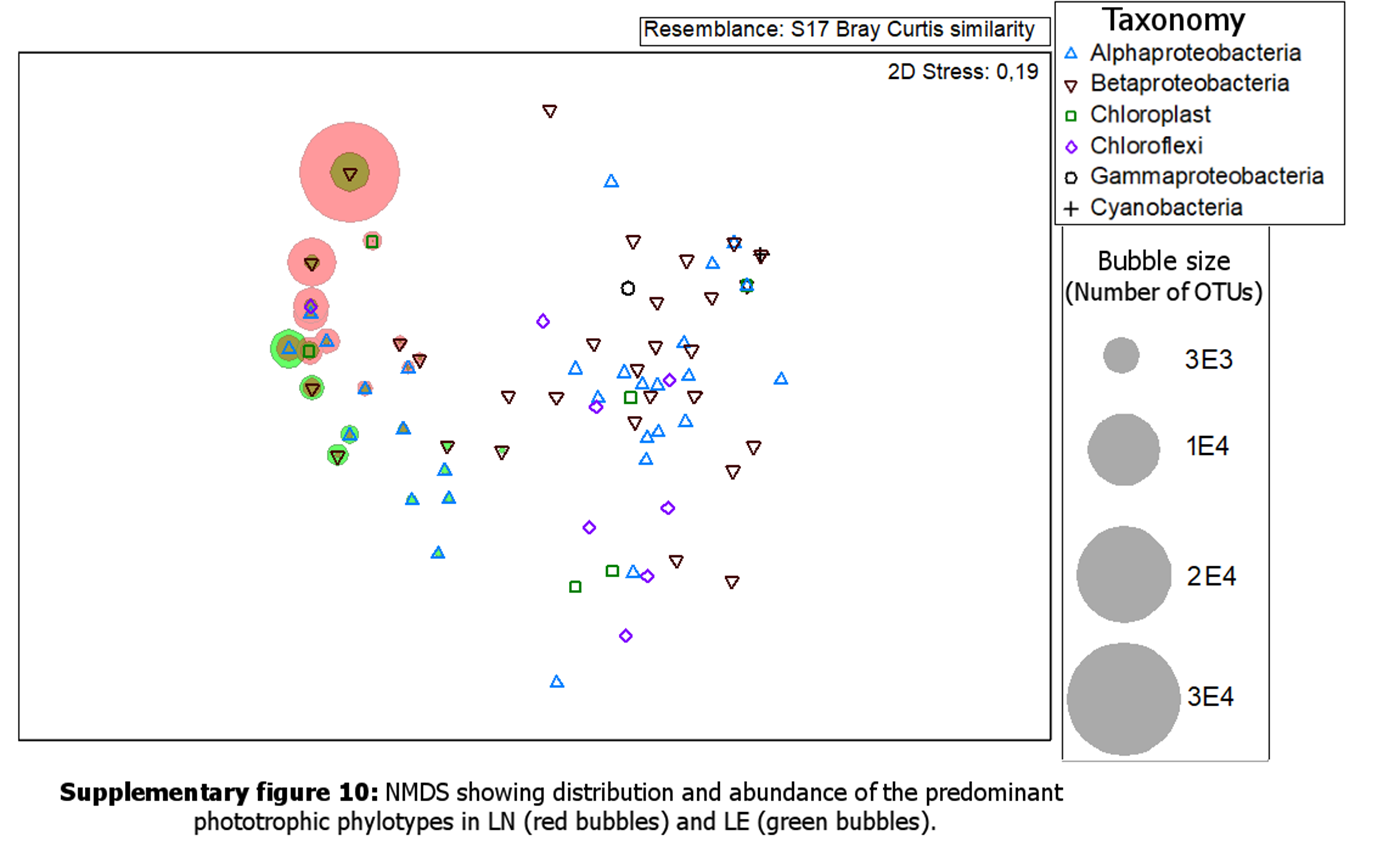

Supplement: Supplementary file 10 [file Image10.TIF]

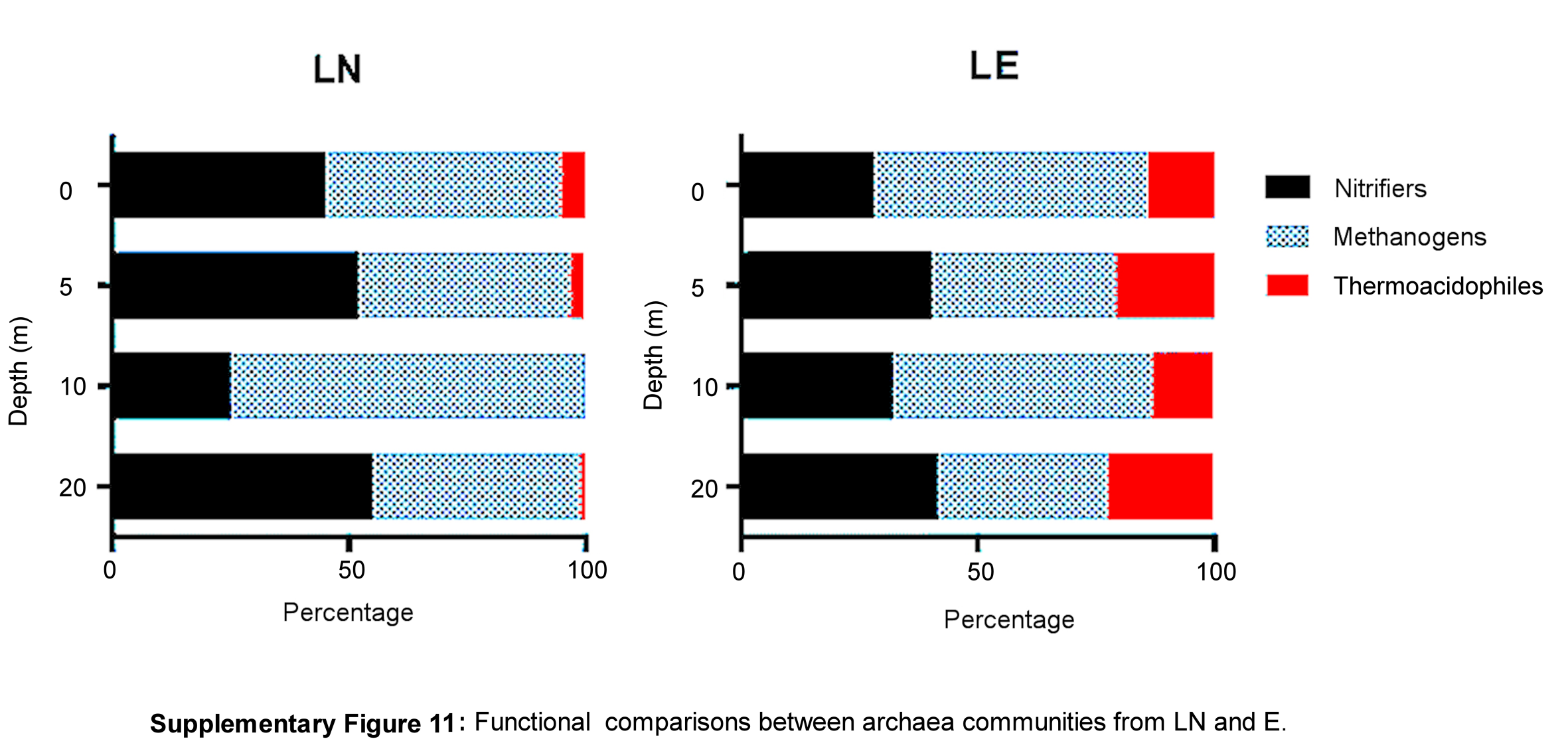

Supplement: Supplementary file 11 [file Image11.TIF]
