# Supplementary material for: Watershed-Induced Limnological and Microbial Status in Two Oligotrophic Andean Lakes Exposed to the Same Climatic Scenario
Source: Front Microbiol. 2018 Mar 5;9:357. doi: 10.3389/fmicb.2018.00357 (PMC5844981; doi:10.3389/fmicb.2018.00357)
Supplement: Supplementary file 12 [file Presentation1.PDF]

Watershed-induced limnological and microbial status in two oligotrophic Andean lakes exposed to the same climatic scenario

Alex Echeverría-Vega[1], Guillermo Chong[2], Antonio E. Serrano[1], Mariela Guajardo[3], Olga Encalada[1], Victor Parro[4], Yolanda Blanco[4], Luis Rivas[4], Kevin C. Rose[5], Mercedes Moreno-Paz[4], José A. Luque[2, 6] , Nathalie Cabrol[7, 8], and Cecilia Demergasso[1]*.

Supplementary Material

The lakes Lo Encañado (LE) and Laguna Negra (LN) are situated in the Andean mountains, 50 km ESE of Santiago, in a region where the geology consists of volcanic and volcanoclastic deposits of Oligocene to Miocene age and Cenozoic plutonic basement (Sernageomin, 2003). The volcanic rocks are of basaltic to andesitic or basaltic nature. The area surrounding LN and LE lakes is volcanically active, being located in the northern part of the Southern Volcanic Zone (SVZ), in Chile. The SVZ is the most active of the three volcanic zones in Chile, with an average of one eruption per year (Stern et al. 2007).

Both lakes are predominantly fed by streams, groundwater and subsurface runoff coming from the Echaurren glacier (33° 35’ S and 70° 8’ W and 3,500 m asl) (see Image 1), and permafrost areas in the watershed. The catchment area of the lakes is a glacial, mountainous region, producing a high amount of eroded and detrital sediments. The amount and type of clastic material change seasonally and annually due to several factors, including changes in rainfall and snow-melting from the glacier.

Climatic changes are triggering variations in the snow cover in the catchment area of Echaurren Glacier. Between 2004 and 2014 the snow cover has been significantly decreased (see Image 1), then also triggering changes in the hydrological system. The LE and LN lakes were hydrologically connected in 2004, but relatively low lake levels led to a hydrological disconnection between the two lacustrine systems as can be seen in the 2014´s image. The views of the oligotrophic lakes are shown in Image 2.

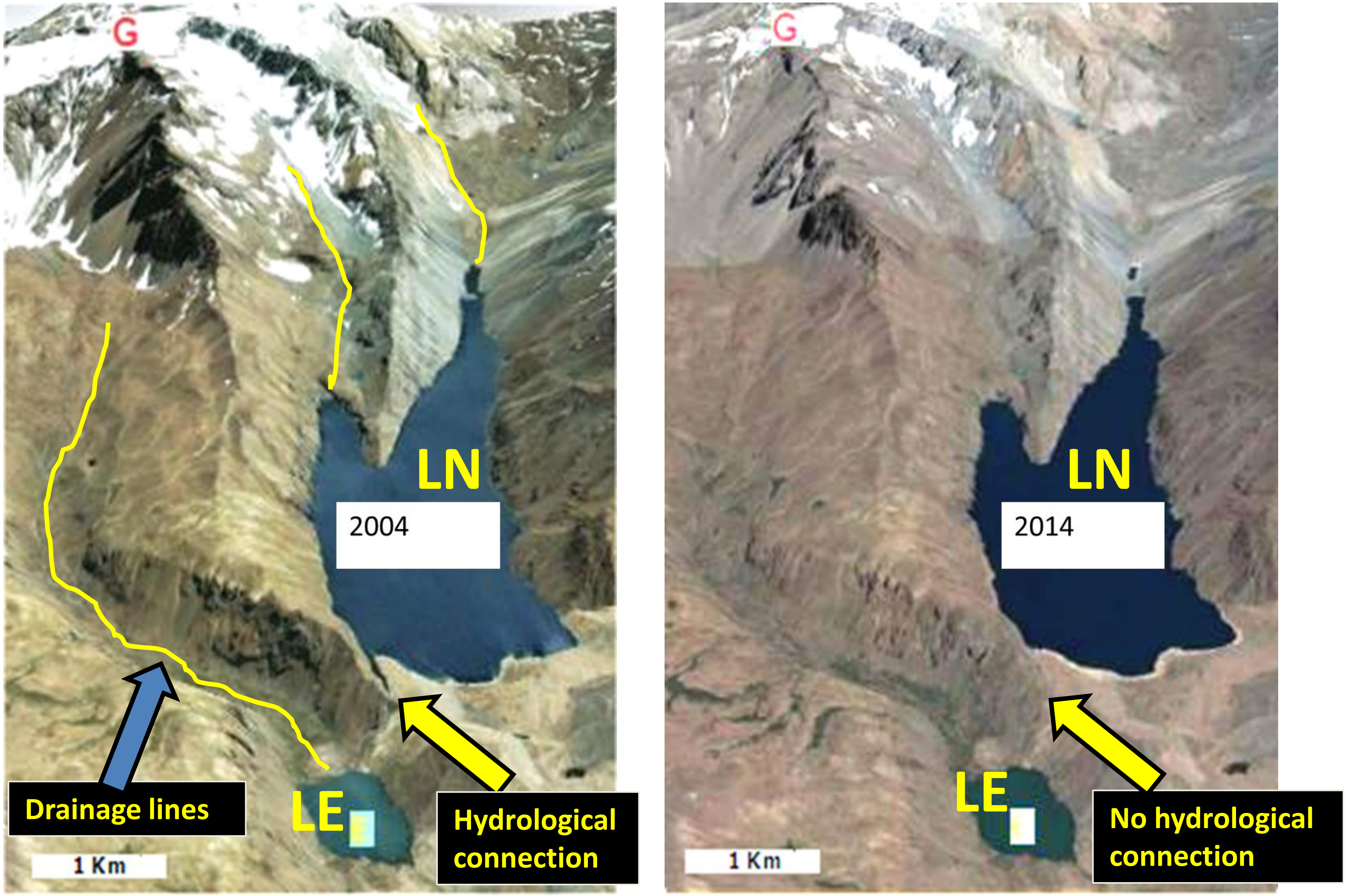

Image 1. Snow cover in the catchment area of Echaurren Glacier (G) between 2004 (left) and 2014 (right). Lo Encañado (LE) and Laguna Negra (LN) were hydrologically connected in 2004, but low lake levels led to hydrological disconnection between the two lacustrine systems (as can be seen in 2014). Images taken from Google Earth.

LN is part of the potable water reservoir of Santiago. The water pH has been previously reported as neutral or slightly alkaline, the mean specific conductance is 131 µS cm−1 and the lake is well oxygenated throughout the water column (Dirección General de Aguas 2014, von Gunten 2009).

Limnological studies were carried out by the national water institution of Chile (Dirección General de Aguas 2014) where limnological data for Laguna Negra was presented. LN showed a stratified column: the shallow epilimnion (relatively warm waters), the intermediate metalimnion (with gradual decreasing in temperature), and the deep hypolimnion (cold waters). In addition, oxygen concentrations increased towards the bottom of the lacustrine system and were associated with the hypolimnion. Regarding electrical conductivity, salinity, Redox potential, turbidity and chlorophyll content, these were constant in depth.

Upstream of the catchment area of Echaurren Glacier, the Lo Encañado River flows through the glacial valley into the LE forming a delta (Pille 2013). Their western and eastern flanks are both very steep, starting directly from the lake shore. LN lies about 200 vertical meters above the eastern flank of the LE. Lake deposits of LE are normally finely laminated, consisting mainly of fine-grained sediments (Pille 2013). The laminations are sometimes disrupted by deposits that are often coarser in grain size and less structured, which denote changes in precipitation and glacier melting. The average sedimentation rate is 1.75 mm/year (Pille 2013, Salvetti 2006). Fining-upwards layers can be found and correlated throughout the lake sediments. They contain high amounts of sand and some pebbles. The range between the large grain size at the base and the fining-upwards trend can only be explained by sub aqueous slides (Pille 2013).

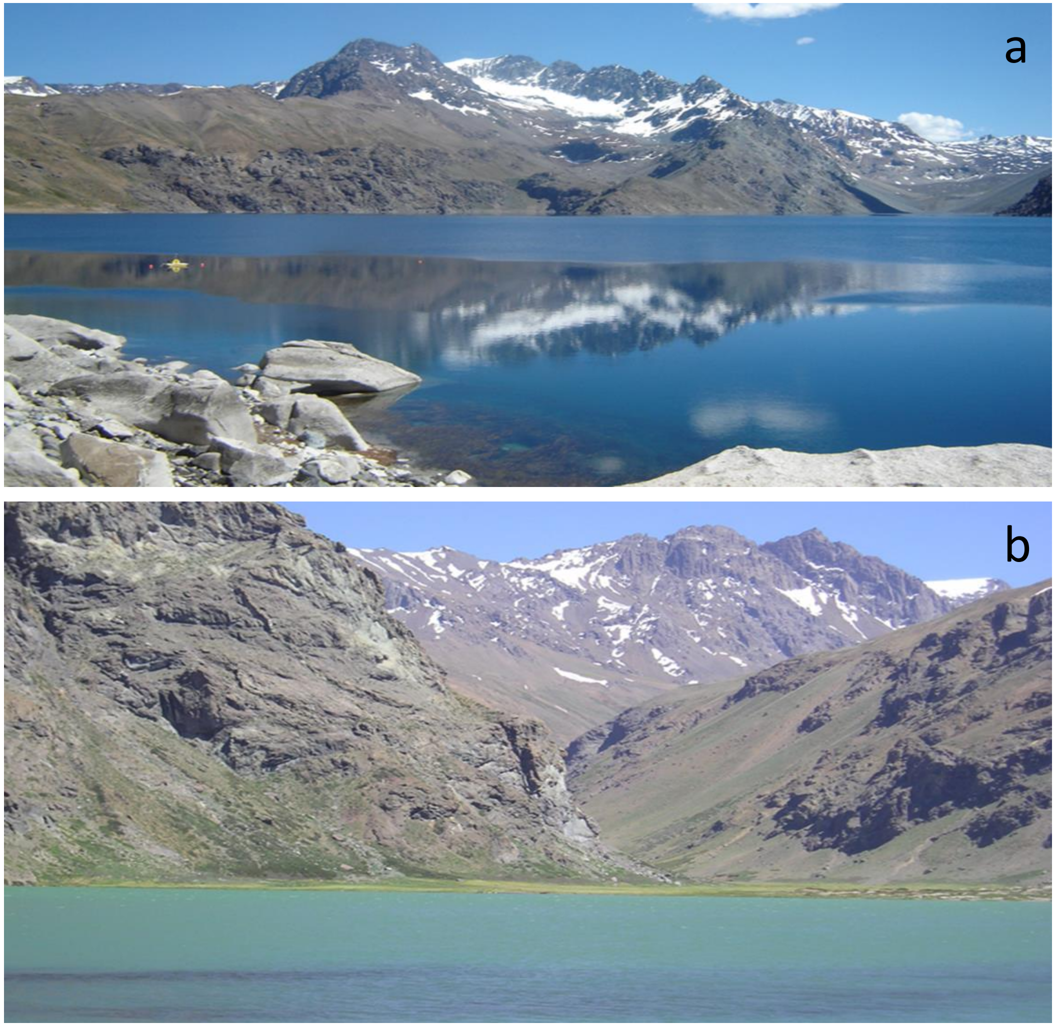

Image 2. Views of the oligotrophic lacustrine systems of LN (a) and LE (b) in December 2012 (photos taken by A. Echeverría).

In addition, Vandenberghe (Vandenberghe 2012) conducted a sedimentological characterization of LN which showed the occurrence of a turbidite at different depths. These turbidites were interpreted as the result of earthquakes. Palaeolimnological and sedimentological investigations at the lakes (Pille 2013, Salvetti 2006, Vandenberghe 2012, von Gunten 2009) demonstrated that turbidities in the lacustrine sediments are easily detected in LE basin.

This observation suggests that the unstable processes associated with earthquakes (turbidites) or associated with run-off erosion processes are more intense in the sub-watershed of LE due to the clastic materials of geological units OM2c and M3i. The geological map of the study area is shown in Image 3. The geological material that constitutes the sub-watershed of LE is made up of Lower to Middle Miocene volcanic complexes that are partly eroded. Specifically, LE lies in the Oligocene to Miocene lava that is basaltic to dacitic in composition, together with epiclastic and pyroclastic rocks (OM2c) and Lower to Middle Miocene volcanic complexes that are partly eroded, consisting of lavas, breccias, domes and pyroclastic deposits (M3i) unit outcrops (Sernageomin 2003).

In contrast, the geological material that constitutes the sub-watershed of LN is mainly made up of hornblendebiotite granodiorite (Msg) and, to a lesser extension, of monzogranites, quartz-bearing monzonites and monzodiorites, which corresponds to the Late Miocene (Image 3). The intensity of erosion processes related to the OM2c and M3i units is higher than the one related to the Msg observed in the sub-watershed of LN. These differences in the watersheds trigger specific limnological and microbial status in the two oligotrophic lakes.

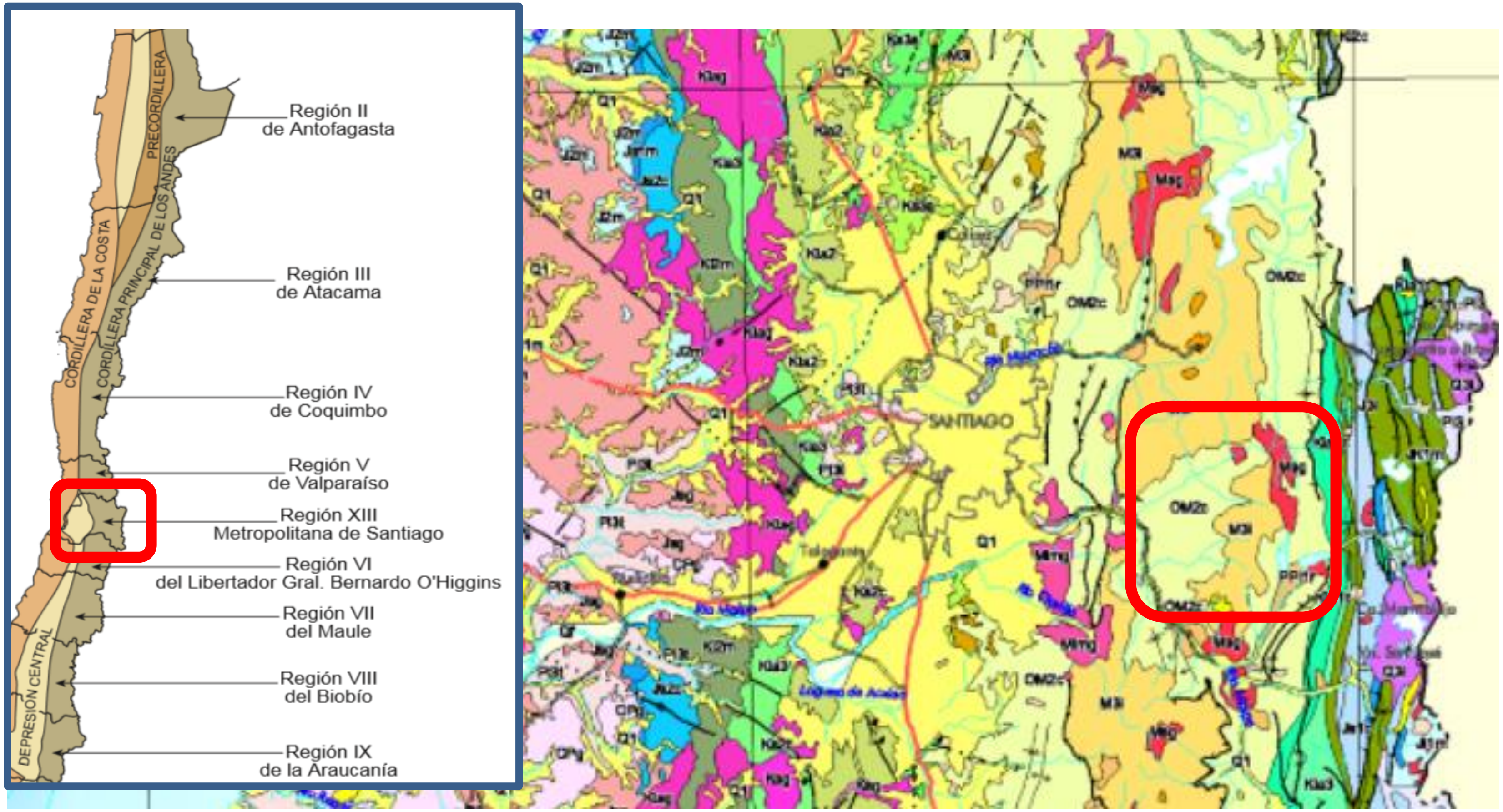

Image 3. Geological map of the study area (Sernageomin, 2003). The erosion and hydrological processes in the catchment areas of LE and LN depend on the identified lithological units (OM2c, M3i and Msg).

Supplementary References

- Dirección General de Aguas (2014). Diagnóstico de la condición trófica de cuerpos lacustres utilizando nuevas herramientas tecnológicas. Santiago, Chile, Ministerio de Obras Públicas, Dirección General de Aguas, Departamento de Conservación y Protección de Recursos Hídricos, Centro de Ecología Aplicada Ltda. S.I.T. N° 347.
- Pille, T. (2013). Event history of the Santiago area (Chile) : the sedimentological archive of Lago Lo Encañado. PhD, Ghent University, Belgium.
- Sernageomin (2003). Mapa Geológico de Chile: versión digital. Servicio Nacional de Geología y Minería, Publicación Geológica Digital, Santiago, Chile.
- Salvetti, C. (2006). Palaeolimnological analysis of lakes in the South Central Andes in Chile. A Case Study of Laguna del Encañado (33°S / 70°W). Master Thesis, University of Bern, Bern.
- Stern, C., Moreno, H., López-Escobar, L., Clavero, J., Lara, L., Naranjo, J., *et al.* (2007). Chilean volcanoes. The Geology of Chile. Moreno T, G. W., The Geological Society, London**:** 147-178.
- Vandenberghe, J. (2012). Late Holocene climate variability in South-Central Chile: a lacustrine record of southern westerly wind dynamics. M.Sc. thesis, Ghent University, Ghent, Belgium.
- von Gunten, L. (2009). High-resolution, quantitative climate reconstruction over the past 1000 years and pollution history derived from lake sediments in Central Chile. PhD of Science in Climate Sciences, Universität Bern, Switzerland.
